# Characterization and T-DNA insertion sites identification of a multiple-branches mutant *br* in *Betula platyphylla* × *Betula pendula*

**DOI:** 10.1186/s12870-019-2098-y

**Published:** 2019-11-12

**Authors:** Rui Han, Chenrui Gu, Ranhong Li, Wendi Xu, Shuo Wang, Chaoyi Liu, Chang Qu, Su Chen, Guifeng Liu, Qibin Yu, Jing Jiang, Huiyu Li

**Affiliations:** 1 State Key Laboratory of Tree Genetics and Breeding, Northeast Forestry University, 26 Hexing Road, Harbin, 150040 China; 2 Institute of Food and Agricultural Sciences, Citrus Research and Education Center, University of Florida, Lake Alfred, FL 33850 USA

**Keywords:** *Betula platyphylla* × *Betula pendula*, Multiple-branches mutant, T-DNA, Insertion and deletion, *BpCOI1*

## Abstract

**Background:**

Plant architecture, which is mostly determined by shoot branching, plays an important role in plant growth and development. Thus, it is essential to explore the regulatory molecular mechanism of branching patterns based on the economic and ecological importance. In our previous work, a multiple-branches birch mutant *br* was identified from 19 *CINNAMOYL-COENZYME A REDUCTASE 1* (*CCR1*)-overexpressed transgenic lines, and the expression patterns of differentially expressed genes in *br* were analyzed. In this study, we further explored some other characteristics of *br*, including plant architecture, wood properties, photosynthetic characteristics, and IAA and Zeatin contents. Meanwhile, the T-DNA insertion sites caused by the insertion of exogenous *BpCCR1* in *br* were identified to explain the causes of the mutation phenotypes.

**Results:**

The mutant *br* exhibited slower growth, more abundant and weaker branches, and lower wood basic density and lignin content than *BpCCR1* transgenic line (OE2) and wild type (WT). Compared to WT and OE2, *br* had high stomatal conductance (Gs), transpiration rate (Tr), but a low non-photochemical quenching coefficient (NPQ) and chlorophyll content. In addition, *br* displayed an equal IAA and Zeatin content ratio of main branches’ apical buds to lateral branches’ apical buds and high ratio of Zeatin to IAA content. Two T-DNA insertion sites caused by the insertion of exogenous *BpCCR1* in *br* genome were found. On one site, chromosome 2 (Chr2), no known gene was detected on the flanking sequence. The other site was on Chr5, with an insertion of 388 bp T-DNA sequence, resulting in deletion of 107 bp 5′ untranslated region (UTR) and 264 bp coding sequence (CDS) on *CORONATINE INSENSITIVE 1* (*BpCOII*). In comparison with OE2 and WT, *BpCOI1* was down-regulated in *br*, and the sensitivity of *br* to Methyl Jasmonate (MeJA) was abnormal.

**Conclusions:**

Plant architecture, wood properties, photosynthetic characteristics, and IAA and Zeatin contents in main and lateral branches’ apical buds changed in *br* over the study’s time period. One T-DNA insertion was identified on the first exon of *BpCOI1*, which resulted in the reduction of *BpCOI1* expression and abnormal perception to MeJA in *br*. These mutation phenotypes might be associated with a partial loss of *BpCOI1* in birch.

## Background

Shoot branching plays an important role in plant growth and development. In gramineous species, such as maize and rice, the number of tillers is directly related to crop biomass, seed yield, and other agronomic traits [1–5]. In woody species, branches limit wood properties and tree architecture, which affect forest vitality and economic benefits [6–8]. Trees with less lateral branches, long internode length, and small tapering are ideal for construction. Comparatively, for shelterbelts and landscaping, multiple-branches trees provide shading, dust absorption, and noise reduction. Therefore, understanding the molecular mechanism of shoot branching development is essential to meet ecological and economical demands for the forest industry.

Two stages are involved in the formation of branches. First, shoot apical meristems (SAMs) form during embryonic development. Then, SAMs in leaf axils differentiate into lateral bud primordia, which develop into lateral buds and form lateral branches [9]. Several factors determine plant shoot branching, including the activity of SAMs, plant hormonal content and distribution, gene expression and environmental factors. For example, light controls the activity of SAM and the growth of preformed leaves to affect the architecture in *Rosa* [10]. The polar transport and gradient distribution of auxin are required for inducing organogenesis, which determines the radial position and size of lateral organs in SAM, subsequently affecting phyllotaxis and inflorescence [11, 12]. Cytokinin is involved in branching and controlling apical dominance. Because of its ability to induce plant cell division, the reduction of cytokinin content in *Arabidopsis thaliana* results in diminished activity of SAM [13, 14]. In addition, genes participating in plant hormone biosynthesis, transduction and SAM formation also correlate with branching [15–20].

Recently, several studies on *Arabidopsis*, *Oryza sativa* and *Zea mays* have been performed to identify and characterize the genes involved in determining branching by using mutants. The findings have led us to better understand the mechanism of plant shoot branching patterns deeply [21–23]. *Betula platyphylla* (birch) is a pioneer and deciduous tree species, which is an important source of pharmaceuticals and biofuels [24, 25]. The completion of genome sequencing of birch makes it possible to explore the gene function using T-DNA insertional mutagenesis in birch [26]. In our previous study, a mutant *br* that exhibited dwarf, multiple-branches, small leaves, and apical buds was identified from *19 BpCCR1* overexpression lines. Results revealed that genes involved in the SAM activity, organogenesis, cell division and differentiation, plant hormone biosynthesis, and signal transduction were differentially expressed by using transcriptome analysis [27]. *br* was identified as one of the *BpCCR1* transgenic lines, while all transgenic lines and WT were transformed with leaves of the same birch line and grown under same conditions. Therefore, all lines had the same genetic background, and the effects of environment and exogeny of *BpCCR1* were excluded. We inferred that the mutation phenotypes of *br* were due to the inserted position of exogenous *BpCCR1* in the genome.

In this study, other characteristics of *br*, including growth traits in the rapid growing stage, wood properties, photosynthetic characteristics, the location and contents of endogenous IAA and Zeatin, were detected compared to OE2 and WT. Meanwhile, the T-DNA insertions of *br* were identified by using whole genome re-sequencing (WGR) analysis. One inserted site located on the CDS of *BpCOI1,* resulting in the reduction of *BpCOI1* expression. *CORONATINE INSENSITIVE 1* (*COI1*) is a signal-switching protein of the Jasmonates (JAs) signaling pathway, which contains F-box motif and leucine-rich repeats. *COI1* is a part of E3 ubiquitin-ligase Skip-Cullin-F-box complex SCF^COI1^ and recruits JASMONATE ZIM domain (JAZ) transcriptional repressor proteins for degradation by the 26S proteasome [28]. The physical interaction of COI1 with the JAZ protein is promoted by an Ile-conjugated form of jasmonic acid (JA-Ile) to serve as a receptor for jasmonate and activates the JA signaling pathway [29, 30]. JAs regulate many processes of plant growth and development, including root growth, stamen development, seed germination, plant senescence and fruit ripening, which also play a key role in plant defense against insect, pathogen attack, and abiotic stresses [31–34]. Among these processes, *COI1* is required for the expression of approximately 84% of 212 JA-induced genes in *Arabidopsis* [35, 36]*.* Our results will help bring new insights to explore the functions of *BpCOI1* and understand molecular basis of plant shoot branching. The achievements obtained in studying the plant architecture of birch will allow us to pave a way for breeding ideal type birch.

## Results

### Growth traits of *br* in rapid growing stage

The tree heights of two-year-old WT, OE2 and *br* were measured every 15 days from May 1st to October 1st, and regressive equations were fitted respectively. The average height of 30 plants from three lines was fitted with a 4-parameter Logistic equation, of which the fitting parameters were higher than 0.95. The height growth process of these three lines exhibited an “S” curve (Additional file 1: Fig. S1, Table 1). Results showed that curve was accurate and reliable to fit the tree height of three lines with 4-parameter Logistic equation and could be used for subsequent height growth analysis and prediction.

**Table 1:** Fitting parameters of Logistic equation for tree height and comparison of growth parameters at rapid stage in two-year-old WT, OE2 and *br*.

Line	*R*^*2*^	Height at the beginning of tree growth *a* (cm)	Height at the end of tree growth *b* (cm)	The beginning of rapid stage *t1* (d)	The end of rapid stage *t2* (d)	Duration of rapid stage RR (d)	The day of maximum growth rate at rapid stage *t0* (d)	The maximum growth rate at rapid stage(cm/d)	Parameters of rapid stage		
									GR (cm)	GD (cm/d)	RRA (%)
WT	0.9987	33.86(a)	174.82(a)	38(b)	89(c)	51(c)	65(c)	1.91 (a)	84.74(a)	1.66(a)	54.62(a)
OE2	0.9977	36.95(a)	162.93(a)	43(a)	98(b)	55(b)	71(b)	1.65 (b)	78.82(b)	1.43(b)	55.56(a)
*br*	09980	21.12(b)	100.00(b)	44(a)	106(a)	62(a)	76(a)	0.96(c)	51.39(c)	0.83(c)	55.13(a)

Different letters indicate significant differences between WT, OE2 and *br* in Duncan-test (*P* < 0.05)

Growth of the three lines began in early May and was stopped growing in mid-September, for a total period of 140 days. The height growth of the *br* (i.e. the measured value after stopping growth) was significantly lower, which was only 57.20% of WT and 61.38% of OE2 (*P* < 0.05). Similarly, the maximum growth rate of *br* was 50.26% of WT and 58.18% of OE2 in the rapid growth stage. Analysis of growth parameters revealed that the beginning and ending times of rapid stage of *br* were relatively delayed, but the repaid stage lasted 1.22 and 1.13 times longer than those of WT and OE2, respectively. The average growth of plant height (GR) and the average daily growth of plant height (GD) of *br* in rapid stage were significantly lower than WT and OE2 (*P* < 0.05). However, no significant difference was found in the ratio of rapid stage growth to annual growth (RRA) (Table 1). The results showed that the beginning of rapid growth of *br* was delayed, and the maximum growth rate and average daily growth at rapid stage were lower during growing season.

### Plant architecture of *br*

**Fig. 1:**
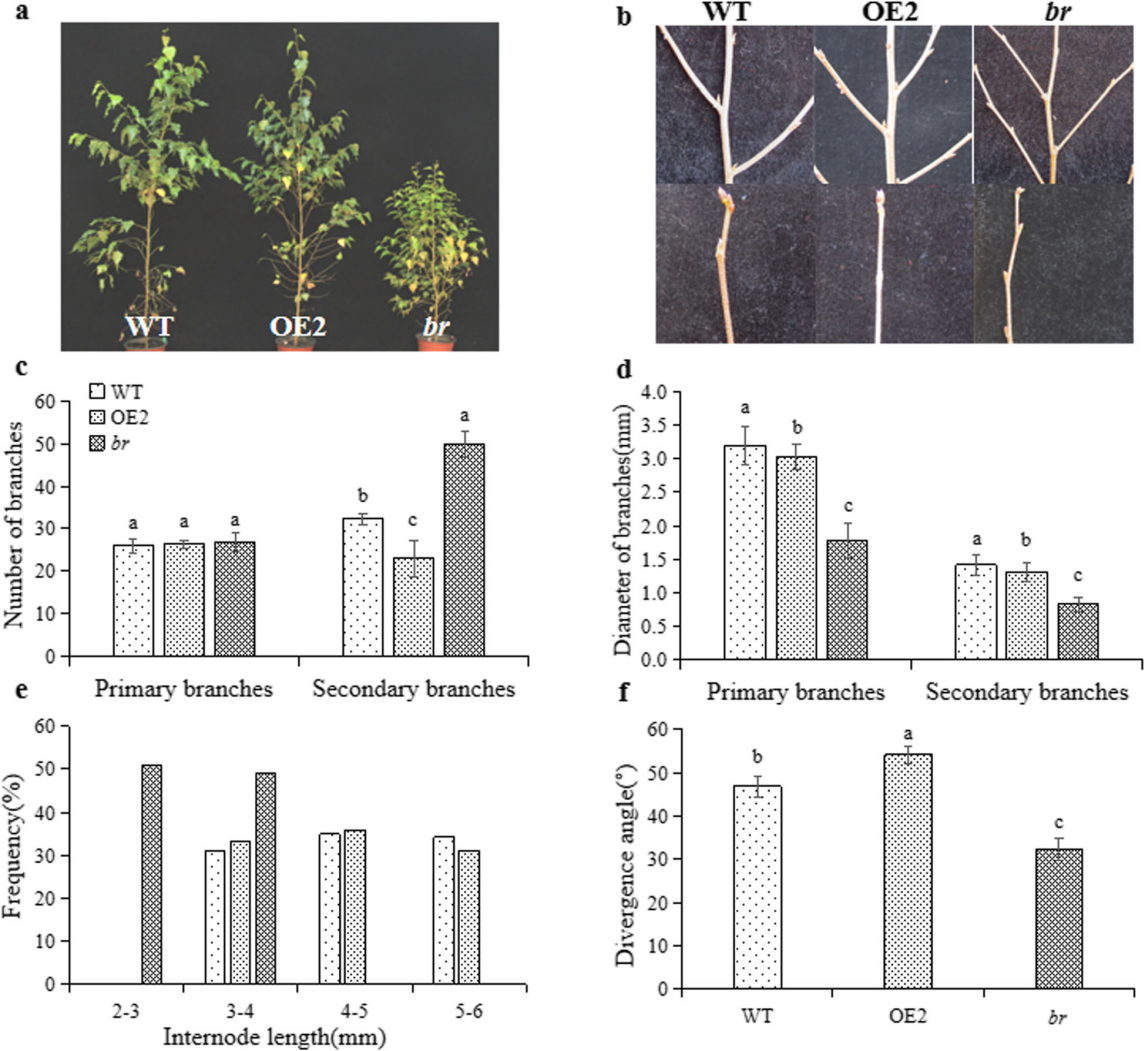
Plant architecture analysis of two-year-old WT, OE2 and *br*. **a** Plant morphology of two-year-old WT, OE2 and *br*. **b** Branches of two-year-old WT, OE2 and *br*. **c**, **d** The number and diameter of primary and secondary branches of three lines. **e** The internodal length of three lines. **f** The divergence angle of three lines. Different letters indicate significant differences between WT, OE2 and *br* in Duncan-test (*P* < 0.05). Values are mean ± standard error of 30 independent replicates per line

The plant architecture of two-year-old *br* was characterized using WT and OE2 as control lines. No significant difference was found in the number of primary branches between *br* and control lines. However, the number of secondary branches of *br* was significantly increased, which was 1.53 times that of WT and 2.16 times that of OE2 (*P* < 0.05) (Fig. 1a, c). Meanwhile, compared to WT and OE2, the diameter of primary and secondary branches was significantly lower, and the branching angle of secondary branches was also significantly smaller in *br* (*P* < 0.05) (Fig. 1b, d, f). The internode length of WT and OE2 ranged from 3 mm to 6 mm. However, the internode length of *br* ranged from 2 mm to 4 mm (Fig. 1e). The results showed that *br* exhibited a shorter internode length, more abundant but weak secondary branches, and narrower branching angle.

### Wood properties of *br*

The wood properties of four-year-old *br* were measured compared to 35S::*BpCCR1* overexpressed transgenic lines (OE2, OE3 and OE4) and WT, including wood basic density, fiber length and width, the contents of lignin, cellulose, holocellulose, hemicellulose, tree height and DBH. No significant differences were found in fiber width and cellulose content between WT and transgenic lines. However, the wood basic density, fiber length, fiber length-width ratio, holocellulose content, and hemicellulose content of the four transgenic lines were lower than those of WT. Among these transgenic lines, *br* exhibited the lowest wood basic density and holocellulose content. The contents of lignin in OE2, OE3 and OE4 were significantly higher than those of WT, while the lignin content of *br* was significantly lower than those of WT and other three transgenic lines. Similarly, the tree height and DBH of *br* were lower compared to the other lines (*P* < 0.05) (Table 2). These results suggested that *br* exhibited a different trend compared to other transgenic lines, in which it displayed a significant reduction of lignin content.

**Table 2:** Multiple comparison of wood qualities and growth traits of tested lines

Line	Basic density (g/cm3)	Fiber length (μm)	Fiber width (μm)	Ratio of fiber length to width	Content of cellulose(%)	Content of hemicellulose (%)	Content of holocellulose (%)	Content of lignin (%)	Tree height (m)	DBH (cm)
WT	0.4141 ± 0.0053a	629.13 ± 70.68a	16.37 ± 2.10b	38.55 ± 3.29a	52.53 ± 0.41ab	26.09 ± 0.09a	78.62 ± 0.49a	11.64 ± 0.60b	3.49. ± 0.17a	15.74 ± 1.00ab
*br*	0.3832 ± 0.0037b	535.97 ± 45.52c	16.14 ± 2.30b	33.62 ± 2.90c	52.27 ± 1.41ab	23.72 ± 0.30 cd	75.99 ± 1.56b	10.95 ± 1.11b	2.17 ± 0.19b	11.17 ± 4.99b
OE2	0.3842 ± 0.0015b	520.05 ± 41.75d	16.42 ± 1.72b	30.86 ± 1.86d	51.57 ± 1.08b	25.19 ± 0.15b	76.68 ± 1.04ab	14.15 ± 0.78a	4.02 ± 0.12a	20.18 ± 2.84a
OE3	0.3850 ± 0.0005b	582.19 ± 65.77b	16.74 ± 1.75b	35.31 ± 2.46b	53.75 ± 0.60a	23.38 ± 0.43d	77.13 ± 0.78ab	13.31 ± 0.51a	3.74. ± 0.55a	18.22 ± 0.06a
OE4	0.3863 ± 0.0060b	511.56 ± 42.01 cd	19.22 ± 1.47a	26.89 ± 1.18e	52.96 ± 0.06ab	23.89 ± 0.10c	76.79 ± 0.05ab	13.49 ± 0.42a	3.86 ± 0.62a	15.07 ± 1.64ab

Wood qualities and growth traits were analyzed using four-year-old WT and transgenic lines. OE2~OE4 indicate 35S::*BpCCR1*△#OE2~35S::*BpCCR1*△#OE4. Different letters indicate significant differences between WT, OE2 and *br* in Duncan-test (*P* < 0.05). Values are mean ± standard error of three measurements from two individual plants per line

### Leaf anatomy, photosynthetic characteristics and chlorophyll fluorescence parameters of *br*

**Fig. 2:**
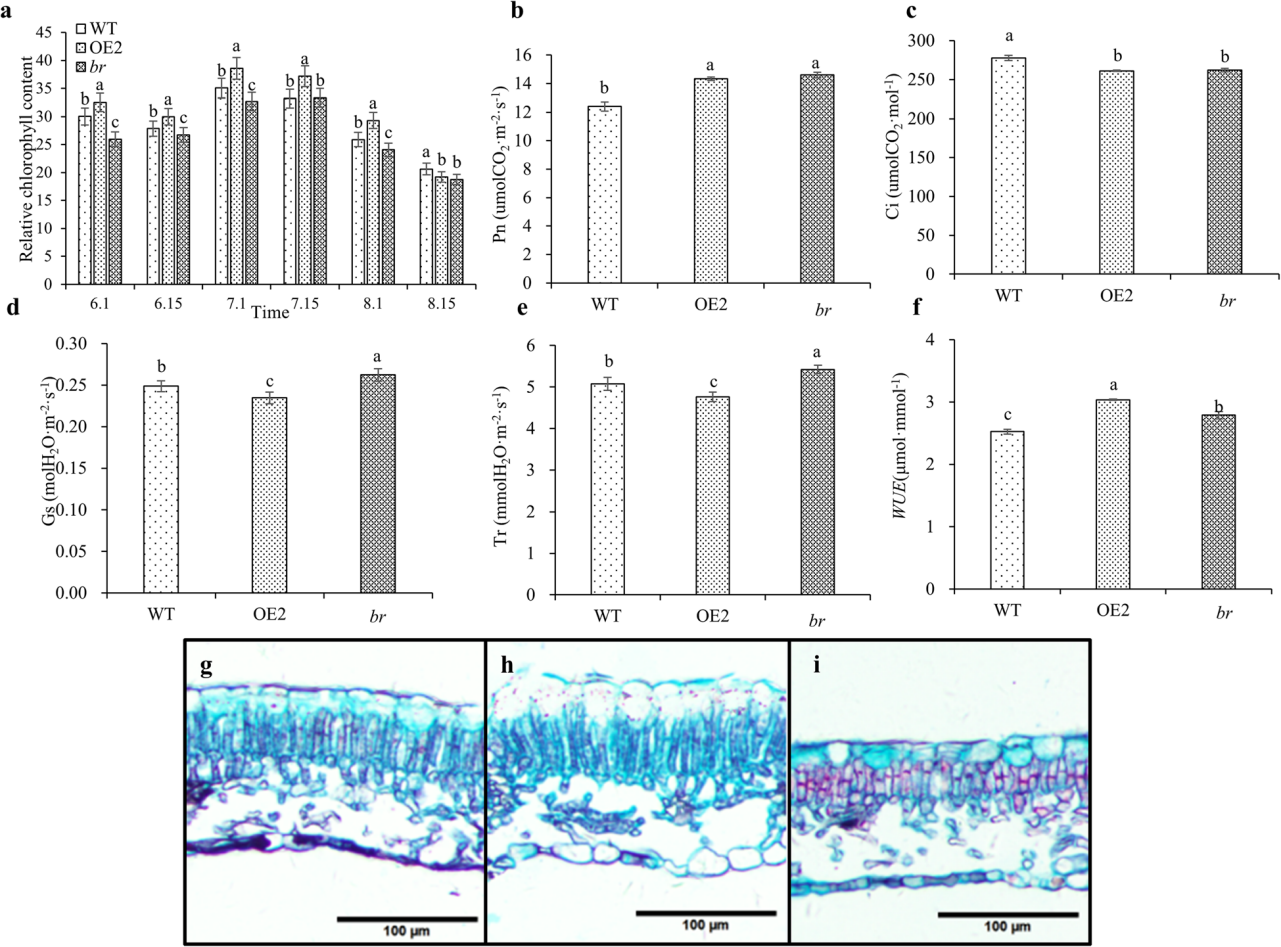
Leaf anatomy, photosynthetic characteristics and chlorophyll fluorescence parameters of two-year-old WT, OE2 and *br*. **a** The relative chlorophyll content of two-year-old WT, OE2 and *br* from June to August. **b**-**f** The net photosynthetic rate (Pn), intercellular CO_2_ concentration (Ci), stomatal conductance (Gs), transpiration rate (Tr) and water use efficiency (*WUE*) of two-year-old WT, OE2 and *br*. **g**-**i** The leaf anatomy of two-year-old WT, OE2 and *br*, bars: 100 μm. Different letters indicate significant differences between WT, OE2 and *br* in Duncan-test (*P* < 0.05). Values are mean ± standard error of nine independent replicates per line

The leaf morphology and size of *br* changed over the study’s timeline, which was indicated in our previous study [27]. To investigate the mechanism of these changes in *br*, the leaf anatomy was observed. Meanwhile, the relative chlorophyll content, photosynthetic, and chlorophyll fluorescence parameters were further examined in two-year-old WT, OE2 and *br*. The thickness of the upper epidermis, lower epidermis, palisade tissue and sponge tissue of *br* were significantly lower than WT and OE2, which were 93.00, 77.79, 64.98, 69.01% of WT and 87.96, 90.22, 70.26, 77.81% of OE2, respectively. The ratio of spongy tissue to leaf thickness was significantly higher, specifically 1.07 times that of WT and 1.09 times that of OE2 (*P* < 0.05) (Fig. 2g-i; Additional file 2: Table S1). The relative chlorophyll content of the three lines showed the same trend from June to August. First, it increased and then decreased, reaching a maximum in early July. The relative chlorophyll content of *br* remained continuously lower compared to WT and OE2 during June to August except on 7.15 and 8.15 (Fig. 2a).

The analysis of photosynthetic characteristics demonstrated that no significant differences were found between OE2 and *br* in intercellular CO_2_ concentration (Ci) and net photosynthetic rate (Pn). The *WUE* of *br* was between that of OE2 and WT (Fig. 2b, c, f). However, stomatal conductance (Gs) and transpiration rate (Tr) of *br* were significantly higher than the other two lines (*P* < 0.05), which were 1.06 times and 1.07 times those of WT, and 1.12 times and 1.14 times those of OE2, respectively (Fig. 2d, e).

The analysis of chlorophyll fluorescence parameters revealed that the maximal photochemical efficiency (*F*_v_*/F*_m_), potential activity (*F*_v_*/F*_0_) and actual photosynthetic efficiency (*Φ*_PSII_) of PSII in *br* were all between those of WT and OE2. However, the non-photochemical quenching coefficient (NPQ) of *br* was lower compared to WT and OE2 (Table 3). All these results suggested that the leaf anatomy of *br* changed, which exhibited the thinned tissue and loosened cells, these changes might be associated with a high stomatal conductance and transpiration rate. In addition, the relative chlorophyll content of *br* was lower than those of WT and OE2, and the lower non-photochemical quenching coefficient demonstrated that the photoprotection capacity of *br* was weak.

**Table 3:** Multiple comparison of chlorophyll fluorescence parameters of two-year-old WT, OE2 and *br*.

Line	*F*_v_*/F*_m_	*F*_v_*/F*_0_	*Φ*_PSII_	*qP*	NPQ
WT	0.73 ± 0.01c	2.62 ± 0.06c	0.48 ± 0.05b	0.86 ± 0.04b	0.73 ± 0.17a
OE2	0.77 ± 0.00a	3.28 ± 0.06a	0.62 ± 0.02a	0.89 ± 0.01a	0.43 ± 0.08b
*br*	0.74 ± 0.00b	2.85 ± 0.05b	0.61 ± 0.02a	0.89 ± 0.02a	0.28 ± 0.06c

Different letters indicate significant differences between WT, OE2 and *br* in Duncan-test (*P* < 0.05). Values are mean ± standard error of nine independent replicates per line

### Distribution and contents of endogenous hormones in *br*

**Fig. 3:**
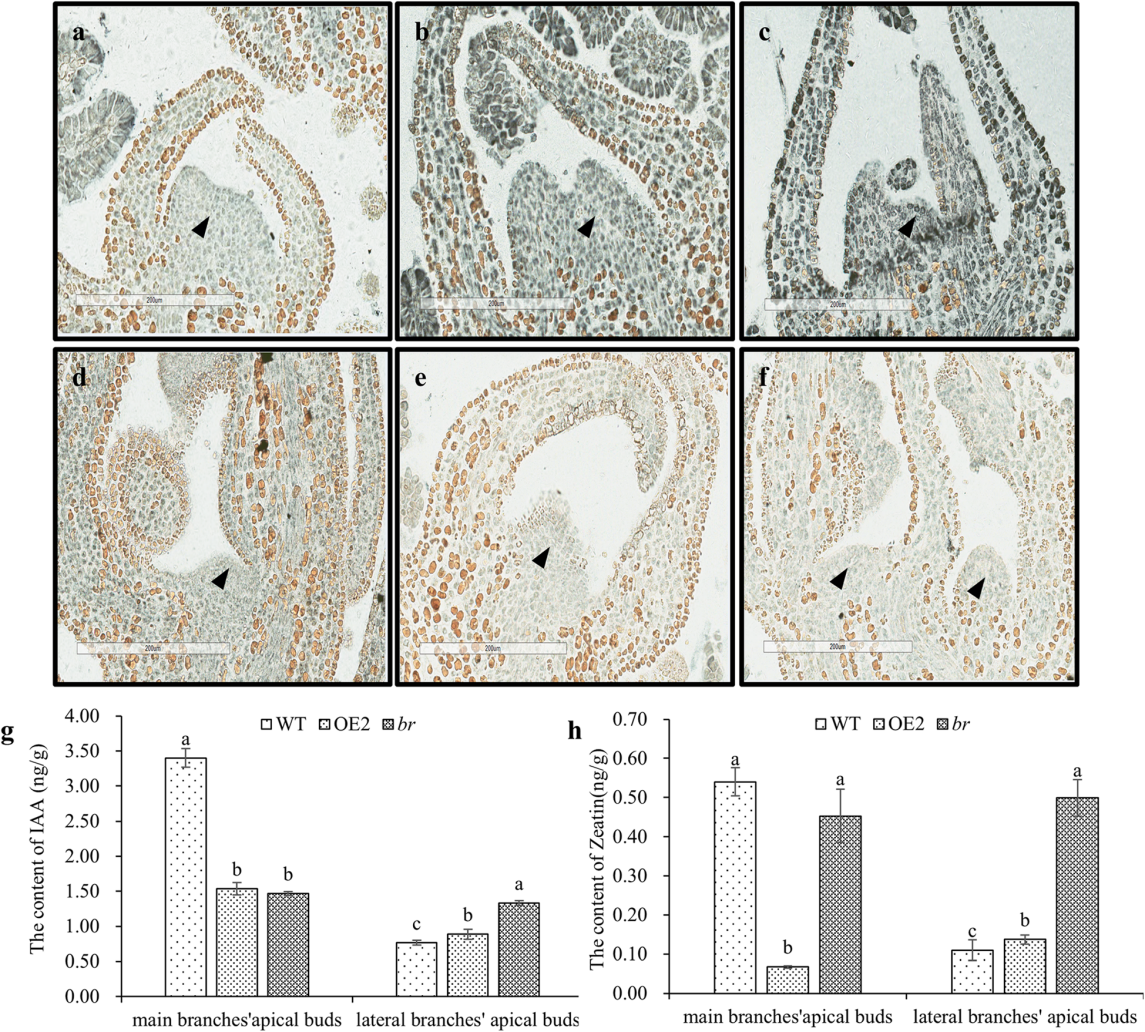
Distribution and contents of endogenous IAA and Zeatin in two-year-old WT, OE2 and *br*. **a**-**c** The location of IAA in lateral branches’ apical buds of two-year-old WT, OE2 and *br*. **d**-**f** The location of Zeatin in lateral branches’ apical buds of two-year-old WT, OE2 and *br*, bars: 200 μm. Arrows in a-f show shoot apical meristems. Stains in a-f indicate the dyeing area by Ag, where endogenous IAA and Zeatin exist. **g**-**h** The contents of endogenous IAA and Zeatin in main and lateral branches’ apical buds of two-year-old WT, OE2 and *br*. Values are mean ± standard error of three technical replicates per line

The distribution and contents of IAA and Zeatin were detected. Immunohistochemical staining was used to localize IAA and Zeatin in lateral branches’ apical buds of WT, OE2 and *br*. The results showed that IAA and Zeatin were distributed in the shoot apical meristem and young leaf tissue cells of all three lines (Fig. 3a-f, Additional file 3: Fig. S2). The contents of IAA and Zeatin in WT, OE2 and *br* were performed by ESI-HPLC-MS/MS. The results revealed that the IAA content ratios of main branches’ apical buds to lateral branches’ apical buds in WT and OE2 were 4.45 and 1.73, for which the Zeatin ratios were 4.91 and 0.49 respectively. However, the IAA and Zeatin content ratios of main branches’ apical buds to lateral branches’ apical buds in *br* were 1.10 and 0.91, which could be approximated to 1 (Fig. 3g, h). Compared to WT and OE2, the ratio of Zeatin to IAA was significantly higher and the ratio of IAA to Zeatin was significantly lower in *br* (*P* < 0.05) (Additional file 4: Fig. S3 a, b). These results demonstrated that no differences were found in IAA and Zeatin distribution between WT, OE2 and *br*, and the contents of IAA and Zeatin in main branches’ apical buds and lateral branches’ apical buds of *br* were similar.

### Identification of T-DNA insertion sites in *br*

WT, *br* and other *BpCCR1* transgenic lines were transformed with leaves of the same birch line and grown under the same conditions, which provided the same genetic background and excluded the effects of environment and exogeny of *BpCCR1*. Therefore, the mutation phenotypes of *br* might be caused by the inserted position of exogenous T-DNA (*BpCCR1*) in genome.

**Fig. 4:**
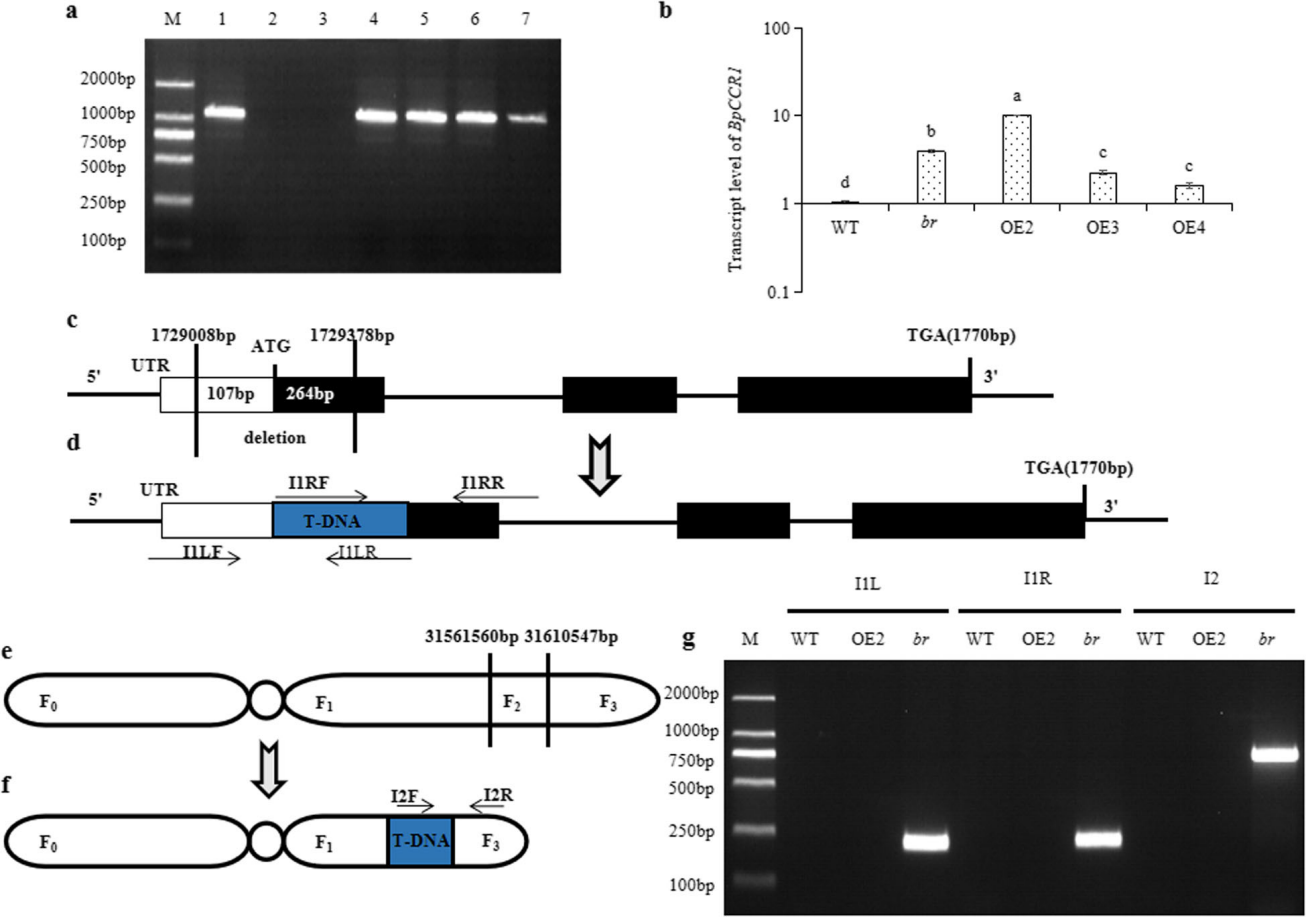
Identification of T-DNA insertion sites in *br*. **a** The detection of *BpCCR1* gene by PCR in 35S::*BpCCR1* plasmid (lane 1), negative control (lane 2), and four-year-old WT (lane 3), *br* (lane 4), 35S::*BpCCR1*△#OE2 (lane 5), 35S::*BpCCR1*△#OE3 (lane 6) and 35S::*BpCCR1*△#OE4 (lane 7) lines. The lane M is DL2000 DNA marker. **b** Transcript level of *BpCCR1* in four-year-old *BpCCR1* over-expression transgenic lines and WT by qRT-PCR. Values are mean ± standard error of three measurements. **c** The insertion site of *br* on Chr5, it shows the normal *BpCOI1* structure. **d** The *BpCOI1* structure after inserting T-DNA. The white boxes are 5’UTR, the black boxes are exons, the lines are introns, and the region separated by vertical lines are deletions. The blue box is insertion. **e** The insertion site of *br* on Chr2, it shows the normal Chr2 structure. **f** The Chr 2 structure after inserting T-DNA. F_0_, F_1_, F_2_ and F_3_ are the chromosome segments. F_2_ indicates the deletion, and blue box is insertion. g: Verification of two insertion sites by PCR. M is DL2000 DNA marker. The first three lanes are I1L, which show the amplification in WT, OE2 and *br*, about the LB on Chr5 based on primers I1LF and I1LR. The middle three lanes are I1R, which display the amplification about the RB on Chr5 in tested lines based on primers I1RF and I1RR. The last three lanes are I2, which indicates the amplification of RB on Chr2 in three lines based on primers I2F and I2R

*BpCCR1* gene was firstly detected as a target gene by polymerase chain reaction (PCR) and quantitative real time polymerase chain reaction (qRT-PCR) in the four transgenic *BpCCR1* overexpression lines (OE2, OE3, OE4 and *br*) to confirm the existence of exogenous T-DNA. Amplified bands about 1000 bp were found in positive plasmid (35S::*BpCCR1*) and transgenic lines, which were not found in negative control and WT (Fig. 4a). Further, the expression of *BpCCR1* was significantly up-regulated in all four transgenic lines (Fig. 4b). These results suggested that the exogenous *BpCCR1* gene was successfully inserted into the genome of birch and could be expressed stably.

T-DNA insertion sites in the genome of *br* were identified by using the second- and third-generation whole genome re-sequencing techniques, respectively. 25,355.31 MB bases were obtained through Illumina Hi Seq 2500 of the second-generation platform. The sequencing depth was 29×, and the Q30 was 92.66%. 98.67% of these data could be mapped to the birch genome. 16,120 MB bases were obtained through PacBio Sequel of the third-generation sequencing platform, of which 99.55% could be mapped to the birch genome. The sequencing depth was 30×, and the average reading length was 8055 bp.

No chromosomal structural mutations including repetition, ectopia, and inversion were found in *br* by Sniffles. 13 suspicious insertion sites were found by BWA, of which 11 corrected-reads without T-DNA sequence were excluded. Two T-DNA insertion sites, 31,561,560–31,610,547 bp on Chr2 and 1,729,008–1,729,378 bp on Chr5, were identified as candidates. There was an insertion of 388 bp vector sequence (from 10,719–11,106 bp) on Chr5, resulting in 371 bp deletion of Bpev01.c0817.g0010 (*BpCOI1*) according to birch reference genome annotation. The deletion fragments included 107 bp 5’UTR and 264 bp CDS upstream sequences of *BpCOI1* (Fig. 4c, d). At 31561560–31610547 bp on Chr2, 8159 bp of PGWB2 vector sequence (from 3967 to 12,125 bp, including 1089 bp of exogenous *CCR1* gene sequence) was inserted, which resulted in 48,988 bp of chromosome deletion (Fig. 4e, f). The comparison results indicated that no known gene was found in the deleted fragment.

The forward and reverse primer sequences were designed based on T-DNA (pGWB2) and the flanking sequence of insertion sites to confirm these two sites via PCR. PCR results showed that no bands were found at WT and OE2, while the length of right-border (RB) and left-border (LB) amplification products at the insertion site of Chr5 in *br* were 200 bp and 207 bp, respectively. The RB amplification on Chr2 was 770 bp (Fig. 4g). The length and resultant sequence of amplification were consistent with our prediction results, indicating that these two sites in *br* were unique and accurate (Additional file 5: DOC 1. a-d). Taken together, based on the same genetic background, the mutation phenotypes of *br* might be associated with the inserted position of exogenous T-DNA (*BpCCR1*) in the genome. The T-DNA could be used as a tag to indicate the insertion sites after confirming the existence of exogenous T-DNA in *br*. Two insertion sites of *br* were identified and verified as 31,561,560–31,610,547 bp on Chr2 and 172,908 bp–1,729,378 bp on Chr5.

### Cloning and bioinformatic analysis of *BpCOI1*

As the T-DNA insertion site on Chr5 was located in the CDS sequences of *BpCOI1* gene, the full open reading frame (ORF) of *BpCOI1* was cloned and sequenced. The results showed that the band was amplified at 1770 bp (Additional file 6: Fig. S4, a). In comparison with birch genome, one base was mutant in *BpCOI1*, which was a synonymous mutation. All three amplifications and sequencings agreed with the above results (Additional file 6: Fig. S4, b). The full length of *BpCOI1* was 1770 bp, encoding a protein of 589 amino acids (Additional file 6: Fig. S4 c). The relative molecular weight of the protein was 66,703.92 Da, and the molecular formula was C_2948_H_4709_N_833_O_862_S_34_ with 9386 atoms in total. 72 positive charge residues (Arg + Lys) and 78 negative charge residues (Asp + Glu) were identified. Of the amino acids, leucine had the relatively highest content, making up 15.1%. COI1 was an acid, unstable and hydrophilic protein, with an isoelectric point of 6.17, theoretical half-life of 30 h, the coefficient of instability of 43.59, aliphatic index of 99.98, and the hydrophilicity evaluation score of − 0.116. A conservative domain of AMN1 from 289 to 438 amino acids and 14 leucine-rich repeats were found (Additional file 6: Fig. S4, d). These results showed that the full-length sequence of *BpCOI1* was identified, and relative bioinformatic analysis of *BpCOI1* was performed.

### Spatiotemporal expression of *BpCOI1* and response to MeJA in *br*

**Fig. 5:**
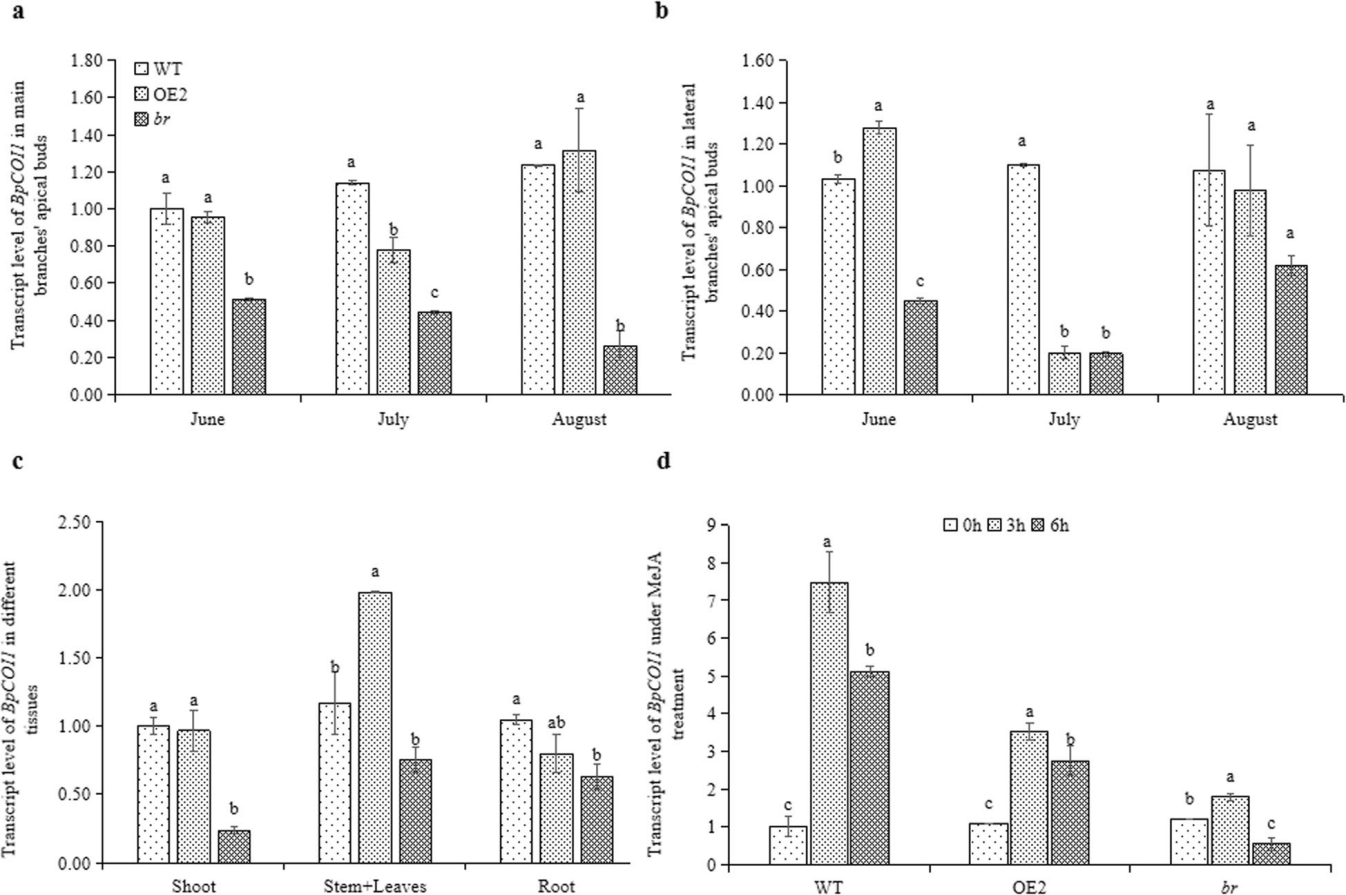
Spatiotemporal expression of *BpCOI1* and response to MeJA in WT, OE2 and *br*. **a** The expression of *BpCOI1* in main branches’ apical buds of two-year-old WT, OE2 and *br.* **b** The expression of *BpCOI1* in lateral branches’ apical buds of two-year-old WT, OE2 and *br*. **c** The expression of *BpCOI1* in shoots, stems (including leaves) and roots of 30-day-old WT, OE2 and *br*. **d** The response to 200 μM MeJA in 30-day-old WT, OE2 and *br*. Different letters indicate significant differences in Duncan-test (*P* < 0.05). Values are mean ± standard error of three measurements

To explore the effect of 371 bp deletion of *BpCOI1* in *br*, the expression of *BpCOI1* in main and lateral branches’ apical buds of two-year-old WT, OE2 and *br* during the June, July and August were detected. The results showed that the expression of *BpCOI1* in main and lateral branches’ apical buds of *br* was lower than those of WT and OE2, but the difference was not significant in lateral branches’ apical buds during July and August (Fig. 5a, b). Similarly, the expression of *BpCOI1* in different tissues of *br* was down-regulated, and significantly lower in apical buds (*P* < 0.05), which was 23.85% of WT and 24.73% of OE2 (Fig. 5c).

*br* exhibited a reduction of *BpCOI1* expression. Therefore, the perception of *br* to Methyl Jasmonate (MeJA) was detected by qRT-PCR. The results showed that compared to WT and OE2, the expression of *BpCOI1* in *br* was up-regulated with a small range under MeJA treatment for 3 h (Fig. 5d). Meanwhile, the response of *br* to MeJA was indicated by the number of rooting seedlings and the average number of main roots under MeJA treatments. No significant differences were discovered in the number of rooting seedlings under 0, 0.5 and 1 μM MeJA treatments. However, this number was significant higher in *br* under 5 and 10 μM MeJA treatments compared to WT and OE2 (Additional file 7: Fig. S5, a, b). Further, the average number of main roots of *br* was also higher than WT and OE2 under 10 μM MeJA treatment (Additional file 7: Fig. S5, c). All these results indicated that the T-DNA insertion site located on *BpCOI1* led to a reduced expression, and abnormal perception of *br* to MeJA.

## Discussion

### Characteristics of mutant *br*

A multiple-branches mutant *br* was identified from *19 BpCCR1* overexpression lines, which exhibited dwarf, small leaves and apical buds, and fertility deficiency. In addition, genes involved in the SAM activity, organogenesis, cell division and differentiation, plant hormone biosynthesis, and signal transduction were differentially expressed in *br* compared WT and OE2 [27]. In this study, other characteristics of *br* were explored, such as the plant architecture. The resulted showed that *br* exhibited multiple-branches, as well as a shorter internode length and narrower branching angle (Fig. 1c, e, f). In addition, the growth traits of *br* in the rapid growing stage were detected. It was found that the beginning of rapid growth of *br* was delayed, and the maximum growth rate and average daily growth rate of *br* were lower compared to those of WT and OE2 (Table 1), which might explain the dwarfing of *br*. Due to changes in the leaf shape and size in *br*, the leaf anatomy was observed in detail. The leaf thickness of *br* decreased, palisade tissues became shorter, and spongy tissue was found to be arranged loosely with large intercellular spacing (Fig. 2g-i, Additional file 2: Table S1). These changes most likely affected the photosynthetic characteristics and chlorophyll fluorescence parameters of *br*. For example, the loosened cells might be associated with a high stomatal conductance and transpiration rate. The wood properties were also analyzed among *br*, WT, and other transgenic lines. Significant differences were observed in the wood density and lignin content between transgenic lines and WT (*P* < 0.05) (Table 2), which was in accordance with previous studies that overexpression of *CCR* could increase the content of lignin in plants [37, 38]. Interestingly, the lignin content of *br* was lower than the other transgenic lines and WT, even if the *BpCCR1* was up-regulated in *br*. However, the reason for this phenomenon remains unclear.

All transgenic lines and WT were transformed with the leaves of the same birch line, and grown under the same conditions, which eliminated the influence of the environment and provided the same genetic background. Therefore, we thought the mutations of *br* might be associated with the inserted position of exogenous *BpCCR1* in the genome.

*Agrobacterium tumefaciens* is a pathogenic factor of crown gall disease, which can transfer T-DNA to plant cells and integrate it into the genome [39, 40]. After transformation, T-DNA can be regarded as a tag to isolate the flanking sequence of insertion sites. Studies have shown that T-DNA is more likely to be integrated in untranslated, transcriptional active or repetitive regions, which makes difficult to exhibit obvious phenotypic changes in mutants [41]. Although reverse PCR, semi-random primer PCR (Tail-PCR), and PCR-walking are frequently used to find T-DNA insertion sites, they have poor specificity, low effective rates, complex operation steps, and need a lengthy operation time [42–44]. Therefore, it is important to establish a simple and efficient method to find T-DNA insertion sites. Recently, the continuous development of whole genome re-sequencing (WGR) technology has demonstrated potential for finding T-DNA insertion sites accurately and efficiently. Several studies have applied this technique for the isolation and identification of mutant genes in rice, soybean, and other mutants [45, 46]. In this study, after confirming the existence of exogenous *BpCCR1*, we employed WGR to find two sites in *br*, in which insertion and deletion occurred in both of them. One site was on Chr2, and no known gene was found on the flanking sequence of this site (Fig. 4e, f). The other site was on Chr5, inserting 388 bp vector sequence and deleting 371 bp fragment. The deleted fragment included 264 bp the first exon of *BpCOI1* and 107 bp 5’UTR, and led to a reduction of *BpCOI1* expression in *br* (Fig. 4c, d; Fig. 5a-c). Taken together, these results suggested that the mutation phenotypes of *br* might be associated with the deletion of *BpCOI1* induced by T-DNA insertion.

### *COI1* participates in plant growth, development, and defense responses

*COI1* participates in almost all the metabolic processes responding to JAs [47]. The *coi1* mutant can abolish JA response, cause male sterility, delay organ abscission and growth rhythm, and weaken JA-regulated defenses [47–50]. In addition, *COI1* is related to meristem arrest and apical dominance, which are governed in an ecotype-dependent manner. The *coi1* mutant displays male sterility, leaf epinasty, dark green leaves, strong apical dominance and enhanced meristem longevity [51]. In our study, there was a reduction of *BpCOI1* expression in *br*, which exhibited abnormal perception to JA response (Fig. 5a-d, Additional file 7: Fig. S5). Therefore, *br* could be regarded as a *coil* mutant that partially deleted the functions of *BpCOI1*. The changes of plant architecture, relative chlorophyll content, and sterility in *br* might be associated with the deletion of *BpCOI1*. However, some phenotypes in birch are different from *Arabidopsis*, we speculate that the gene expression profiles of tree species are more complicated than herbaceous species, and the gene functions in different species are distinct.

### Hormones determine branching development

Classical topping, grafting, and exogenous hormones treatment confirm that auxins and cytokinins are important hormones involved in shoot branching [52, 53]. Auxins are synthesized at the primary shoot apex and young leaves, from which auxins are transported in the polar transport steam and inhibit shoot branching [54]. While roots are the major site of cytokinins synthesis, cytokinins move acropetally from the roots and promote the growth of axillary meristem [52]. Cytokinins are proposed as a second messenger, which mediates the action of auxins in controlling the apical dominance. In this study, the distribution and contents of IAA and Zeatin were detected. Immunohistochemical staining of IAA and Zeatin revealed no significant difference in the distribution of the two hormones between WT, OE2 and *br* (Fig. 3a-f), while the contents of IAA and Zeatin in main and lateral branches’ apical buds in *br* were about the same (Fig. 3g-h). Compared to WT and OE2, in *br*, the ratios of IAA to Zeatin in both main and lateral branches’ apical buds were significantly lower, while the ratios of Zeatin to IAA were significantly higher (Additional file 4: Fig. S3). Previous studies have demonstrated that exposing callus cultures to a low auxin-to-cytokinin promotes shoot development [55]. A low CTK/IAA ratio usually produces few shoots, whereas a high CTK/IAA ratio has the reverse effect [56, 57]. Therefore, we proposed that an increased number of branches in *br* might be due to the higher ratio of Zeatin to IAA.

A recent study has indicated the existence of a novel hormonal signal strigolactones (SLs), which are good candidates for the second messenger of auxins, and can also act in a regulatory loop with auxins to control branching [58]. Further, Zheng et al. and Nakamura et al. found that brassinolides (BRs) and gibberellins (GAs) signaling pathways participated in SLs signaling pathways through *BES1* and *SLR1*, respectively, and regulate plant branching together [59, 60]. However, it remains unknown whether JAs interact with the above signaling network through *COI1* to determine shoot branching in birch. The molecular mechanism of JA-*BpCOI1*-mediated branching of birch need to be further analyzed.

## Conclusions

In summary, mutation phenotypes of plant architecture, wood properties, photosynthetic characteristics and IAA and Zeatin content ratio of main branches’ apical buds to lateral branches’ apical buds were exhibited in *br*. Two T-DNA insertion sites were identified in *br*. One site, on the first exon of *BpCOI1*, resulted in the reduction of *BpCOI1* expression and abnormal perception to MeJA, while the second site displayed no known gene. Overall, the results suggested that the mutation phenotypes of *br* might be associated with a partially lost function of *BpCOI1* in birch.

## Methods

### Plant material

The birch (*Betula platyphylla* × *B. pendula*) multiple-branches mutant *br*, *BpCCR1* overexpression lines OE2, OE3, OE4, and wild type birch (WT) were originally obtained from Northeast Forestry University (Harbin, China) [61]. Four-year-old plants were grown in pots with dimensions of 55 cm × 55 cm, and two-year-old plants were grown in pots with dimensions of 35 cm × 35 cm under natural conditions at the breeding base of the Northeast Forestry University, Harbin, China. *br* has been identified as a mutant from *BpCCR1* overexpression lines, and OE2 was used for excluding the function of *BpCCR1* [27].

### Determination of wood properties and growth characteristics

Four-year-old *br*, OE2, OE3, OE4 and WT were cut off as 2-cm block segments at 10 cm above the ground for use as samples. The 3-mm pieces from the pith outward were immersed in a mixture of 10% nitric acid and 10% chromic acid, then washed with water until the rinse solution was neutral according to the Frey method [62]. The length and width of fibers were measured under optical microscope (ZEISS, Germany). The values of fiber length and width were taken as the average of 30 individual fibers.

The contents of cellulose, holocellulose, hemicellulose and lignin of four-year-old *br*, OE2, OE3, OE4 and WT were determined by an ANKOM 2000i automatic fiber analyzer (Ankom, USA). Hemicellulose, cellulose and lignin were obtained as the residues of digestion of the neutral detergent fiber (NDF). Cellulose and lignin remained after digestion of the acid detergent fiber (ADF). After an acid washing lignin (ADL) treatment, residues were placed in a muffle furnace to obtain ash. The values of contents were calculated as the average of three measurements from two individual plants, as follows:

1 $$ \mathrm{Hemicellulose}\%=\mathrm{NDF}\%-\mathrm{ADF}\%, $$

2 $$ \mathrm{Holocellulose}\%=\mathrm{NDF}\%\hbox{-} \mathrm{ADL}\%, $$

3 $$ \mathrm{Cellulose}\%=\mathrm{ADF}\%\hbox{-} \mathrm{ADL}\% $$

4 $$ \mathrm{Lignin}\%=\mathrm{ADL}\%\hbox{-} \mathrm{Ash}\% $$

Four-year-old *br*, OE2, OE3, OE4 and WT were cut off as 2-cm wood blocks to analyze wood basic density, according to the national standard GB1933–2009 [63]. The volume was measured by the drainage method, and the samples were baked at 80 °C to determine the constant weighs. Each value of basic density was the average of three measurements from two individual plants. The basic density was calculated as follows:

5 $$ \mathrm{Basic}\ \mathrm{density}\ \mathrm{of}\ \mathrm{wood}\ \left(\mathrm{g}/{\mathrm{cm}}^3\right)=\mathrm{dry}\ \mathrm{weight}/\mathrm{volume}\ \mathrm{of}\ \mathrm{blocks}\ \mathrm{at}\ \mathrm{water}\ \mathrm{saturation}, $$

From May to October 2017, the plant height of two-year-old WT, OE2 and *br* was measured. After plants became dormant, the numbers and diameters of the primary and secondary branches, and angles of branches were measured. Each value of these characteristics was taken as the average of 30 individual plants.

### Observation on leaf anatomy

The fourth leaves of two-year-old WT, OE2 and *br* were cut off as 3 × 5 mm blocks equidistant from central veins. Then, they were fixed in FAA (formaldehyde, glacial acetic acid, and 50% ethyl alcohol, V:V:V = 1:1:18) for 24 h, dehydrated with an ascending series of ethanol (70, 80, 85, 95, and 100%), cleared with xylene, and embedded in paraffin. 10-μm thick sections were made on an HM 340E microtome (Thermo, USA), then stained by safranine and fast green (Sigma, USA). Leaf anatomy of the samples was examined and photographed using Olympus DP26 (Olympus, Japan). The thicknesses of leaves, epidermal cells, palisade tissues and spongy tissues were measured using a CellSens Entry (Olympus, Japan). Each thickness value was taken as the average of three measurements from three individual plants. The ratio of palisade to spongy tissue, tightness of palisade tissue, and looseness of spongy tissue were calculated as follows:

6 $$ \mathrm{Ratio}\ \mathrm{of}\ \mathrm{palisade}\ \mathrm{to}\ \mathrm{spongy}\ \mathrm{tissue}=\mathrm{Palisade}\ \mathrm{tissue}\ \mathrm{thickness}/\mathrm{Sponge}\ \mathrm{tissue}\ \mathrm{thickness} $$

7 $$ \mathrm{Tightness}\ \mathrm{of}\ \mathrm{palisade}\ \mathrm{tissue}\ \left(\mathrm{CTR}\right)=\mathrm{Palisade}\ \mathrm{tissue}\ \mathrm{thickness}/\mathrm{Leaf}\ \mathrm{thickness} $$

8 $$ \mathrm{Tightness}\ \mathrm{of}\ \mathrm{sponge}\ \mathrm{tissue}\ \left(\mathrm{SR}\right)=\mathrm{Sponge}\ \mathrm{tissue}\ \mathrm{thickness}/\mathrm{Leaf}\ \mathrm{thickness} $$

### Determination of photosynthetic and chlorophyll fluorescence parameters

From June to August 2017, the relative chlorophyll contents of two-year-old WT, OE2 and *br* were detected by a SPAD-502 chlorophyll meter (Chincan, China) every 15 days. Each value of the relative chlorophyll content was the average of 30 individual plants.

Net photosynthetic rate (Pn), stomatal conductance (Gs), intercellular CO_2_ concentration (Ci), and transpiration rate (Tr) of fourth leaves in two-year-old WT, OE2 and *br* were measured using a Li-co-6400 portable photosynthetic analyzer (LI-COR, USA) on sunny days precisely at 9:00 am. Relative air humidity was about 50%, and leaf temperature was about 25 °C. The light intensity was 1400 μmol·m^− 2^·s^− 1^. Each value of these parameters was the average of nine individual plants. The instantaneous water use efficiency (*WUE*) was calculated as follows:

9 $$ WUE=\mathrm{Pn}/\mathrm{Tr} $$

The chlorophyll fluorescence parameters were measured by a pulsed chlorophyll fluorescence meter PAM-2500 (WALZ, Germany) after 20 min of dark adaptation. The maximum photochemical efficiency (*F*v/*F*m), potential activity (*F*v/*F*0), actual photosynthetic efficiency (*Φ*_PSII_), photochemical quenching coefficient (*qP*), and non-photochemical quenching coefficient (NPQ) of PSII were recorded, respectively. Each value of these parameters was the average of nine individual plants.

### Immunohistochemical staining and endogenous hormone determination

Lateral branches’ apical buds of two-year-old WT, OE2 and *br* were pre-fixed with 2% EDC (Sigma, USA), then fixed with 4% paraformaldehyde (Coolaber, China) overnight at 4 °C. Then, the samples were washed with phosphate buffer (0.2 M, pH 7.2) (Sinopharm Chemical Reagent Co., Ltd., China), dehydrated with gradient ethanol (70, 85, 95 and 100%), excessed with 1/2 xylene + 1/2 ethanol, cleared with xylene and embedded in paraffin for 24 h. 10-μm thick sections were made on an HM 340E microtome (Thermo, USA), and then dry at 45 °C for 2 h.

The immunolocalization of IAA and Zeatin followed the procedure proposed by Gao et al. with some modifications [64]. Slices were incubated with 0.1% trypsin (Beytome, China) for 10 min at 37 °C, and then incubated in a blocking solution [0.05 M Tris buffer pH 7.6 (TBS) (Sigma, USA), 0.3% (v/v) Triton X-100 (BioFrox, Germany), 10% (v/v) normal goat serum (Beytome, China), and 5% (w/v) bovine serum albumin (BSA) (Beytome, China)] for 30 min. Primary IAA antibody (mouse monoclonal, Sigma, USA) and primary Zeatin antibody (rabbit polyclonal, Agrisera, Sweden) were diluted and used to incubate for 2 h at 37 °C (anti-IAA antibody was diluted with antibody diluent (Bioss, China) at 1:1, and anti-Zeatin antibody was diluted at 1:5). Subsequently, sections were washed briefly in regular salt rinse solution [0.05 M Tris buffer pH 7.6 (TBS), 0.3% (v/v) Triton X-100, 5% (w/v) BSA], blocked out for 15 min, and incubated for 1 h at 37 °C with the gold-labeled goat anti-mouse IgG (IAA, Sigma, USA) and the gold-labeled goat anti-rabbit IgG (Zeatin, Sigma, USA) (secondary IAA and Zeatin antibody were diluted with antibody diluent at 1:50). Samples were rinsed three times with TBST [0.05 mol/L Tris buffer pH 7.6 (TBS), 0.3% (v/v) TritonX-100], twice times with double distilled water, 5 min for each time. After washing, sections were submitted to the silver-enhancement reaction in a staining solution [0.1 M citrate buffer (pH 3.5) (Sinopharm Chemical Reagent Co., Ltd., China), 1.7% (w/v) hydroquinone (Bodi Chemical Co., Ltd., China), 0.1% (w/v) silver nitrate (Binhaiyinpeng Chemical Co., Ltd., China), 5% (w/v) gelatin (Sigma, USA)] for 10 min at 25 °C. Then, sections were rinsed twice in double distilled water later, dehydrated, mounted, observed and photographed using Aperio ImageScope (Leica, USA).

IAA and Zeatin in main and lateral branches’ apical buds of two-year-old WT, OE2 and *br* were extracted from 6 plants in each line using the isopropanol/water/hydrochloric acid extraction method. IAA and Zeatin were quantitatively separated by ESI-HPLC-MS/MS with an AB Qtrap6500 (Agilent, USA), which was performed by Zoonbio Biotechnology (Nanjing, China). IAA and Zeatin standard products were purchased from Sigma (MO, USA). The value of these contents was taken as the average of three measurements.

### Identification of T-DNA insertion sites

The total DNA and RNA of four-year-old *br*, OE2, OE3, OE4 and WT were extracted from leaves as templates using the DNA quick Plant system (Tiangen Biochemical Technology, China) and total RNA fast isolation kit (BioTeke corporation, China). According to ORF of *BpCCR1*, the forward primer: 5′-ATGGGATTCGGGGGGGCCGAAG-3′, and reverse primer: 5′-CTAATTCATTGTAACAATCGGAGTTC-3′ were designed to detect the exogenous *BpCCR1* gene, WT and H_2_O were used as the negative control, and pROKII-CCR as the positive control. cDNA was synthesized from 1 μg RNA of tested lines using a ReverTra Ace® qPCR RT Kit (Toyobo, Japan) according to the manufacturer’s instructions. The expression of *BpCCR1* was examined using qRT-PCR, which was performed on a 7500 real-time PCR system (Darmstadt, Germany) with SYBR® Green PCR master mix (Toyobo, Japan). The forward primer was 5′-AGCATGTGCGAGAACACCATC-3′, the reverse primer was 5′-ACTCATCACTCCAGCAGCCA-3′. *Bp18S* rRNA was used as an internal reference gene with forward primer 5′-ATCTTGGGTTGGGCAGATCG-3′ and reverse primer 5′- CATTACTCCGATCCCGAAGG-3′. The amplification system and procedure were carried out according to Huang et al. [65]. The relative expression level of genes was calculated using the 2^-△△CT^ method [66]. Each value of the expression was taken as the average of three measurements.

To find the insertion sites of T-DNA, *br* genome was re-sequenced using high-throughput second-generation sequencing (BioMarker, China) and third-generation sequencing (Novogene, China) using the total DNA of four-year-old *br*. Sniffles was used to process third-generation data and find chromosome structural mutations, including duplication, ectopia and inversion [67]. The second-generation pair-end reads were mapped to the reference genome and modified pGWB2 (T-DNA) sequence using BWA [26, 68]. The pair-end reads, including both T-DNA and chromosome sequences, were viewed with samtools 1.9 (https://github.com/samtools/samtools) and screened. Their locations were predicted as suspicious insertion sites. Concurrently, third-generations long-reads were mapped to the reference genome by minialign (https://github.com/ocxtal/minialign). Long-reads which were mapped to the suspicious insertion sites, then extracted as target reads. The corrected-reads were obtained by combining the second- and third-generation sequencing data using proovread [69–72]. The corrected-reads without the T-DNA sequence were excluded, and those containing the genome and T-DNA sequence were selected and analyzed using NCBI-blast 2.

The primers from pGWB2 (I1LR:5′-CACTACGTGAACCATCACCCA-3′, I1RF:5′-CGTCCGCAATGTGTTATTAAGTTGTC-3′, I2F: 5′-CTATCGTGGCTGGCCACGA-3′) and birch genome (I1LF:5′-TCCTATTCCGACGATCTCCACC-3′, I1RR:5′-CTGACGATCATT.

CGCCGGAAG-3′, I2R:5′-CCTGGCAAGGTCTCGTCAT-3′) were designed to confirm T-DNA insertion sites with the total DNA of WT, OE2 and *br* as templates. Furthermore, *BpCOI1* was cloned, using cDNA of WT (forward primer was 5′-ATGGAGGATCGGAACGTGAGC-3′, reverse primer was 5′-CTACGCGGCAACTAAGGCTTC-3′, according to CDS). The primers were synthesized by Genray Biotechnology (Shanghai, China). The PCR products were sequenced, which was performed by Boshi Biotechnology (Harbin, China).

### Spatiotemporal expression of *BpCOI1* and treatment with MeJA

Main and lateral branches’ apical buds of two-year-old WT, OE2 and *br* were picked up in mid-June, mid-July, and mid-August. Then they were treated with liquid nitrogen to extract the total RNA for analyzing the temporal expression of *BpCOI1* using qRT-PCR. WT, OE2, and *br* were cultured in a woody plant medium (WPM) for 30 days, then the total RNA of was extracted from the apical buds, stems (including leaves), and roots of each line to analyze the tissue-specific expression of *BpCOI1* using qRT-PCR.

Thirty-day-old WT, OE2 and *br* with identical growth were treated with 200 μM MeJA (Sigma, USA). The total RNA was extracted to analyze the expression of *BpCOI1* using qRT-PCR after treatment for 0, 3, and 6 h. *Bp18S* rRNA was used as an internal reference gene. The primers of *BpCOI1* were designed within deletion fragments. The forward primer was 5′-ATGCCGTACATCCACGACTC-3′, the reverse primer was 5′-CTGACGATCATTCGCCGGAAG-3′. Each value of the expression was taken as the average of three measurements.

Nine plants of WT, OE2 and *br* with identical growth were grown at woody plant medium with 0, 0.5, 1, 5 and 10 μM MeJA for 30 days. The number of rooting seedlings and the average number of main roots were measured. The roots of WT, OE2 and *br* were examined and photographed using an Olympus DP26 (Olympus, Japan). Each value of the expression was the average of three independent replicates.

### Data analysis

SPSS16.0 was used to perform data analysis. Curve fitting (Matlab) was used to fit the equation. ProtParam was used to analyze the physical and chemical properties of *BpCOI1*, and ORF software was employed to find open reading frames, Conserved domains were analyzed with NCBI-Conserved Domains.

## Supplementary information

**Additional file 1: Figure S1.** a: The tree height of two-year-old WT, OE2 and *br* from May 1st to October 1st. b, c and d: Fitted equations of WT, OE2 and *br*.

**Additional file 2: Table S1.** Multiple comparison of leaf microstructure index of two-year-old WT, OE2 and *br*. Different letters indicate significant differences between WT, OE2 and *br* in Duncan-test (*P* < 0.05). Values are mean ± standard error of three measurements from three individual plants per line.

**Additional file 3: Figure S2.** Negative control of immunohistochemical staining without antibody, bar: 200 μm. Arrow shows SAM.

**Additional file 4: Figure S3.** The contents of endogenous hormones in two-year-old WT, OE2 and *br*. a: The ratio of IAA to Zeatin in main and lateral branches’ apical buds of two-year-old WT, OE2 and *br*. b: The ratio of Zeatin to IAA in main and lateral branches’ apical buds of two-year-old WT, OE2 and *br*. Different letters indicate significant differences between WT, OE2 and *br* in Duncan-test (*P* < 0.05). Values are mean ± standard error of three technical replicates per line.

**Additional file 5: DOC 1.** The analysis of flanking sequence in *br.* a: The sequence of T-DNA (pGWB2); b: The sequence of I1L; c: The sequence of I1R; d: The sequence of I2. The sequences in red are mapped to pGWB2, and the sequences in green are mapped to the birch genome in b, c and d. The sequence without a shadow indicates misalignment caused by T-DNA insertion in c.

**Additional file 6: Figure S4.** Cloning and bioinformatic analysis of *BpCOI1* gene. a: The amplification of *BpCOI1* by PCR with cDNA of WT as the template, M is DL2000 DNA marker, arrow indicates the size of fragment; b: Alignment of reference and cloned *BpCOI1,* red box indicates the base mutant; c: The analysis of open reading frames; d: The analysis of conservative domains.

**Additional file 7: Figure S5.** The responses of WT, OE2 and *br* to MeJA. a: The roots of 30-day-old WT, OE2 and *br* under 0, 0.5, 1, 5 and 10 μM MeJA treatments, bars: 1 mm; b: The number of rooting seedlings of 30-day-old WT, OE2 and *br*; c: The average number of main roots in 30-day-old WT, OE2 and *br*. Different letters indicate significant differences between WT, OE2 and *br* in Duncan-test (*P* < 0.05). Values are mean ± standard error of three independent replicates per line.

## Data Availability

All data generated or analyzed during this study are included in this published article and its supplementary information files.

## Abbreviations

ADF: Acid detergent fiber
ADL: Acid detergent lignin
BRs: Brassinolides
BSA: Bovine serum albumin
*CCR1*: *CINNAMOYL-COENZYME A REDUCTASE 1*
CDS: Coding sequence
Chr 2 and Chr 5: Chromosome 2 and chromosome 5
Ci: Intercellular CO_2_ concentration
*COI1*: *CORONATINE INSENSITIVE 1*
DBH: Diameter at breast height
*F*_v_*/F*_0_: Potential activity of PSII
*F*_v_*/F*_m_: Maximal photochemical efficiency of PSII in the dark
GAs: Gibberellins
GD: The average daily growth rate of plant height at rapid stage
GR: The average growth of plant height at rapid stage
Gs: Stomatal conductance
IAA: Auxin
JAs: Jasmonates
JAZ: JASMONATE ZIM
LB: Left border
MeJA: Methyl Jasmonate
NDF: Neutral detergent fiber
NPQ: Non-photochemical quenching coefficient
OE2, OE3, OE4: 35S::*BpCCR1* overexpressed transgenic lines
ORF: Open reading frames
PCR: Polymerase chain reaction
Pn: Net photosynthetic rate
PSII: Photosystems II
*qP*: Photochemical quenching coefficient
qRT-PCR: quantitative real time polymerase chain reaction
RB: Right border
RRA: The ratio of rapid stage growth to annual growth
SAM and SAMs: Shoot apical meristem and shoot apical meristems
SLs: Strigolactones
TBS: Tris buffer
Tr: Transpiration rate
UTR: Untranslated region
WGR: Whole genome re-sequencing
WPM: Woody plant medium
WT: wild type
*WUE*: Water use efficiency
*Φ*_PSII_: Actual photosynthetic efficiency
