# Supplementary figures and images for: Characterization and T-DNA insertion sites identification of a multiple-branches mutant br in Betula platyphylla × Betula pendula
Source: BMC Plant Biol. 2019 Nov 12;19:491. doi: 10.1186/s12870-019-2098-y (PMC6852751; doi:10.1186/s12870-019-2098-y)

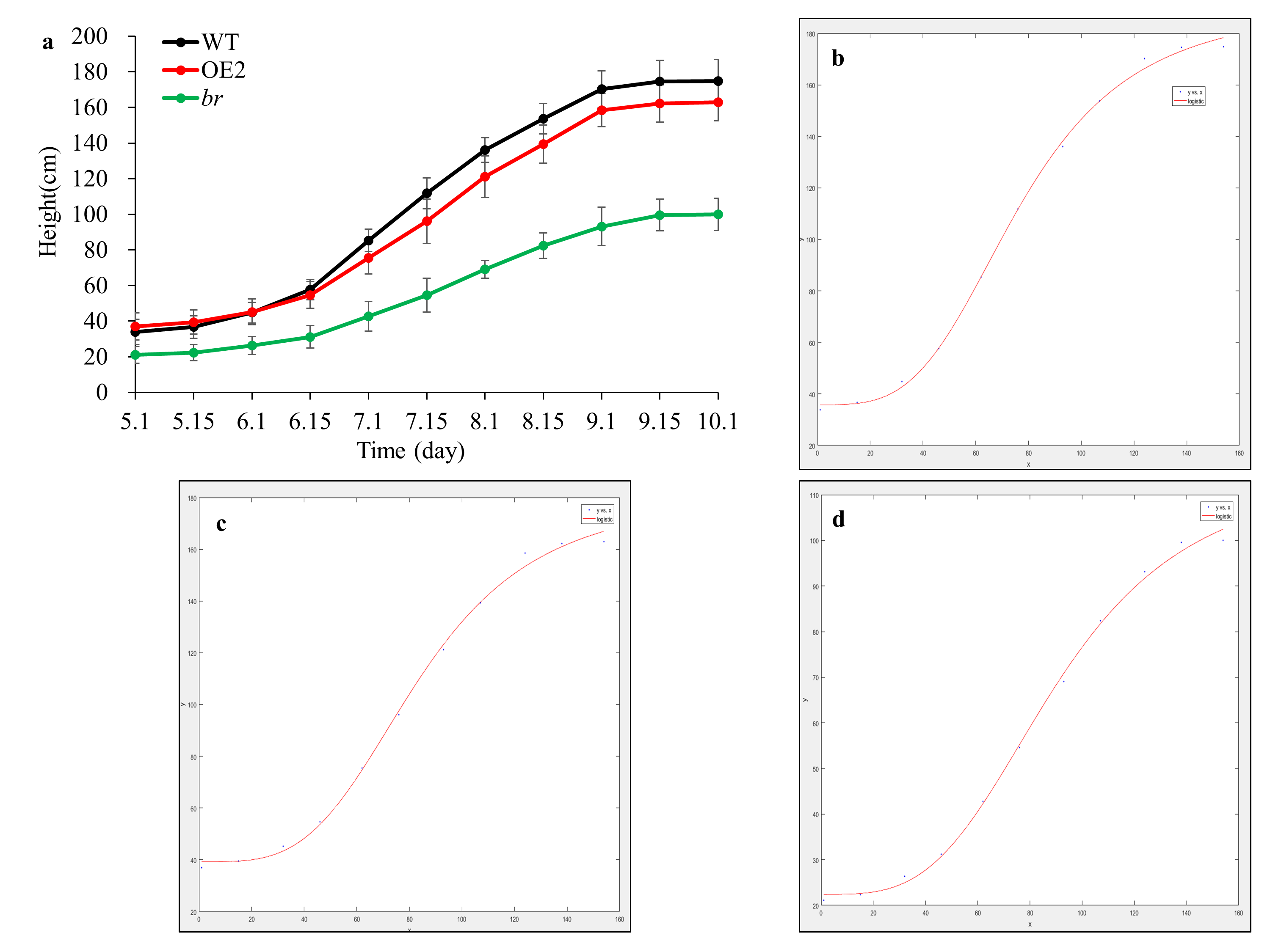

Supplement: Supplementary file 1 — Additional file 1: Figure S1. a: The tree height of two-year-old WT, OE2 and br from May 1st to October 1st. b, c and d: Fitted equations of WT, OE2 and br. [file 12870_2019_2098_MOESM1_ESM.tif]

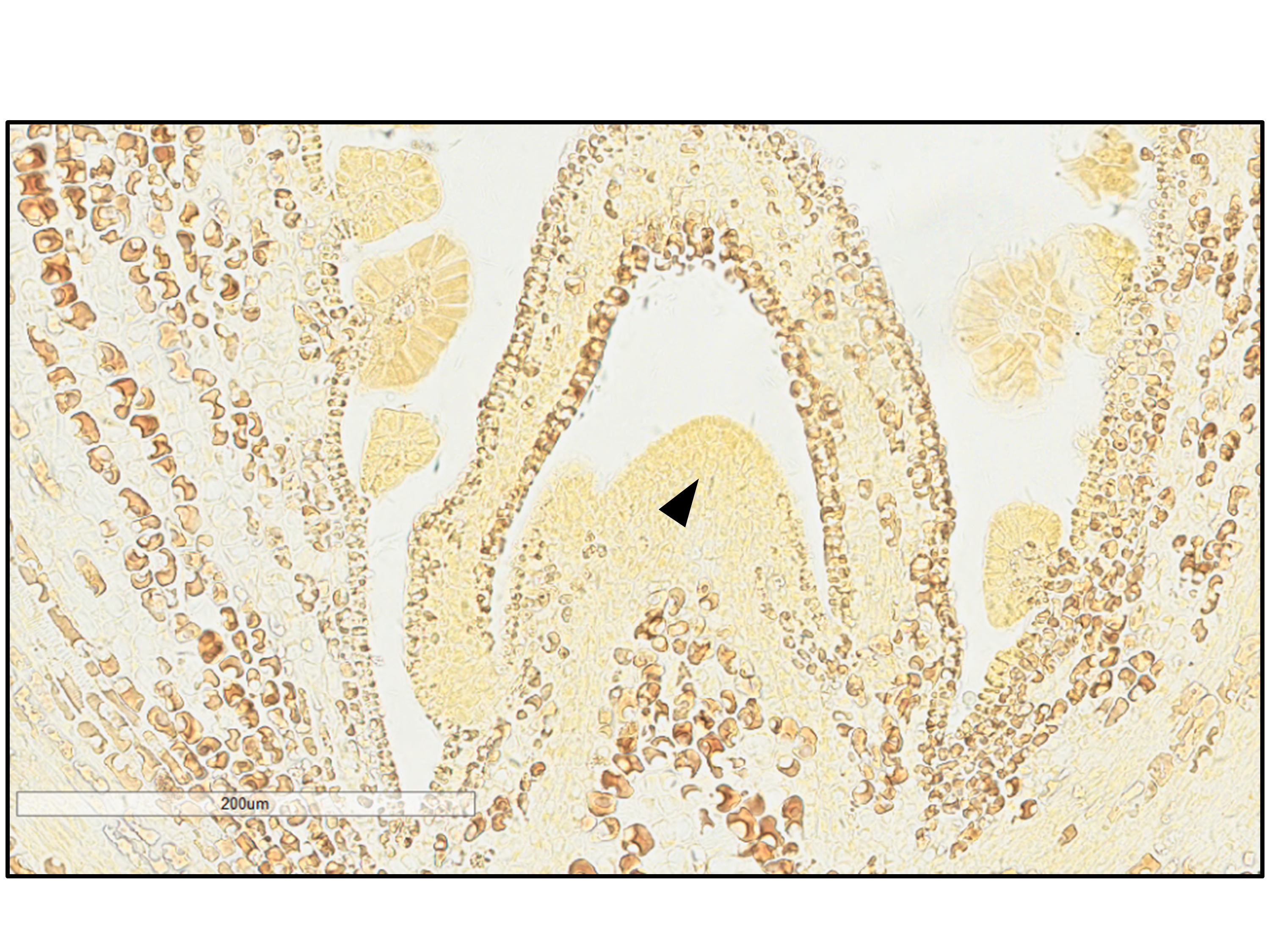

Supplement: Supplementary file 3 — Additional file 3: Figure S2. Negative control of immunohistochemical staining without antibody, bar: 200 μm. Arrow shows SAM. [file 12870_2019_2098_MOESM3_ESM.tif]

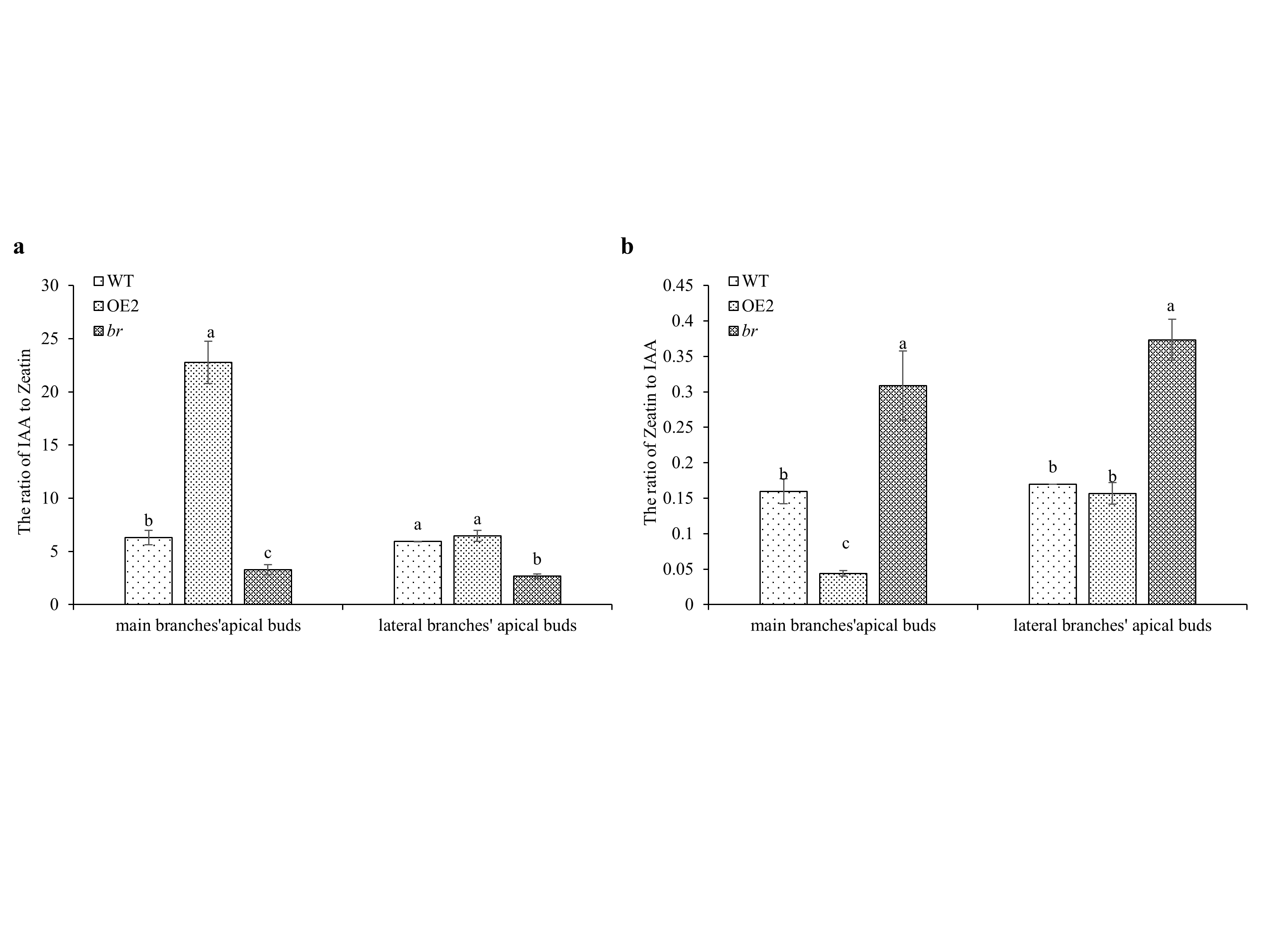

Supplement: Supplementary file 4 — Additional file 4: Figure S3. The contents of endogenous hormones in two-year-old WT, OE2 and br. a: The ratio of IAA to Zeatin in main and lateral branches’ apical buds of two-year-old WT, OE2 and br. b: The ratio of Zeatin to IAA in main and lateral branches’ apical buds of two-year-old WT, OE2 and br. Different letters indicate significant differences between WT, OE2 and br in Duncan-test (P < 0.05). Values are mean ± standard error of three technical replicates per line. [file 12870_2019_2098_MOESM4_ESM.tif]

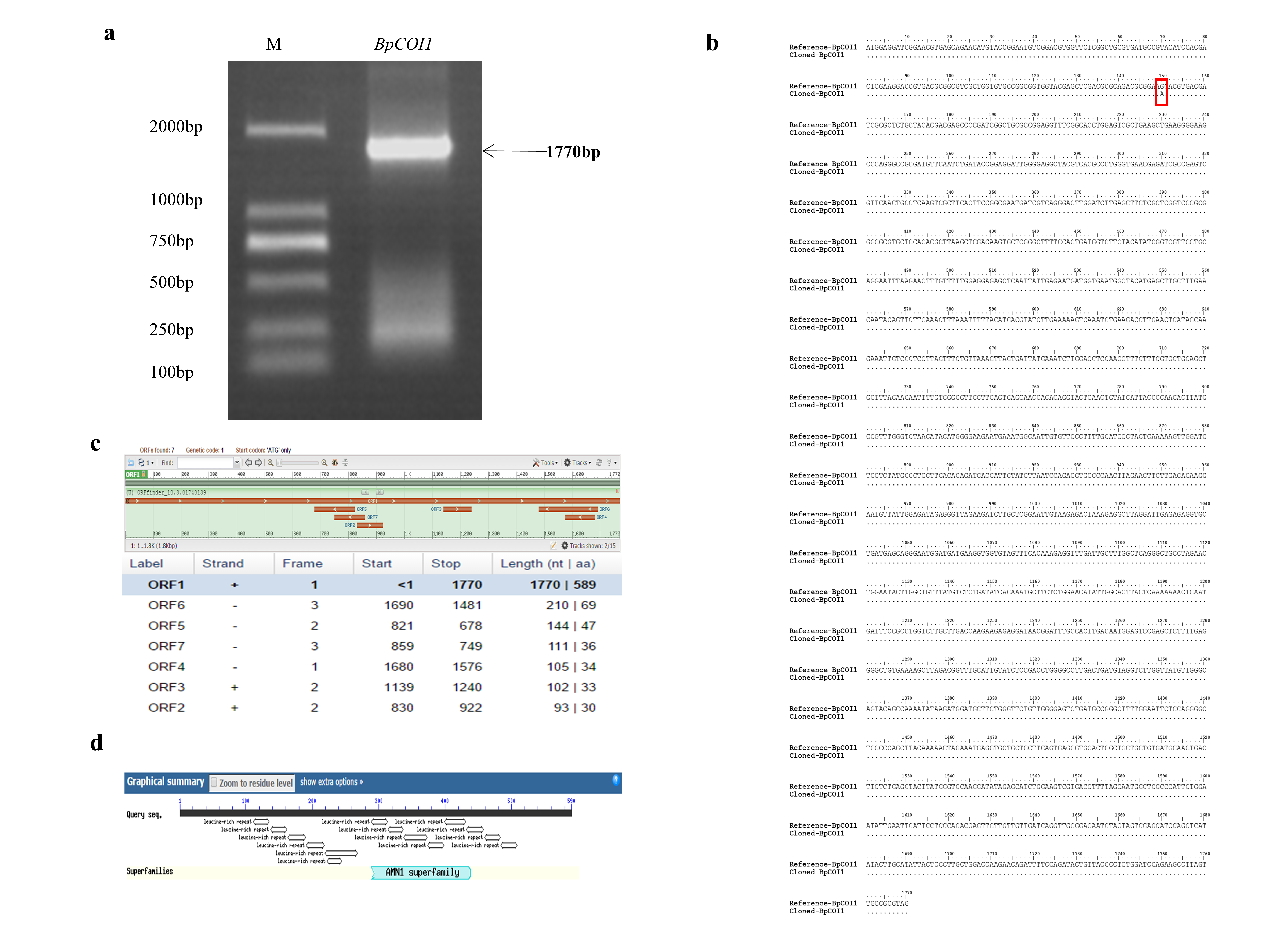

Supplement: Supplementary file 6 — Additional file 6: Figure S4. Cloning and bioinformatic analysis of BpCOI1 gene. a: The amplification of BpCOI1 by PCR with cDNA of WT as the template, M is DL2000 DNA marker, arrow indicates the size of fragment; b: Alignment of reference and cloned BpCOI1, red box indicates the base mutant; c: The analysis of open reading frames; d: The analysis of conservative domains. [file 12870_2019_2098_MOESM6_ESM.tif]

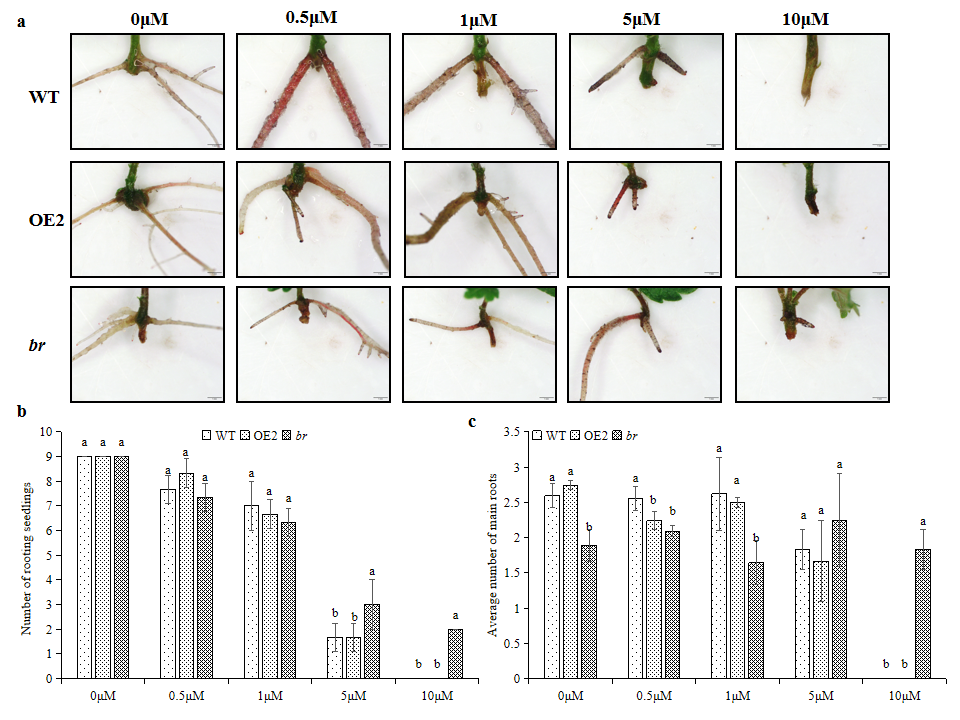

Supplement: Supplementary file 7 — Additional file 7: Figure S5. The responses of WT, OE2 and br to MeJA. a: The roots of 30-day-old WT, OE2 and br under 0, 0.5, 1, 5 and 10 μM MeJA treatments, bars: 1 mm; b: The number of rooting seedlings of 30-day-old WT, OE2 and br; c: The average number of main roots in 30-day-old WT, OE2 and br. Different letters indicate significant differences between WT, OE2 and br in Duncan-test (P < 0.05). Values are mean ± standard error of three independent replicates per line. [file 12870_2019_2098_MOESM7_ESM.tif]
