# Supplementary material for: Characterization and T-DNA insertion sites identification of a multiple-branches mutant br in Betula platyphylla × Betula pendula
Source: BMC Plant Biol. 2019 Nov 12;19:491. doi: 10.1186/s12870-019-2098-y (PMC6852751; doi:10.1186/s12870-019-2098-y)
Supplement: Supplementary file 2 — Additional file 2: Table S1. Multiple comparison of leaf microstructure index of two-year-old WT, OE2 and br. Different letters indicate significant differences between WT, OE2 and br in Duncan-test (P < 0.05). Values are mean ± standard error of three measurements from three individual plants per line. [file 12870_2019_2098_MOESM2_ESM.doc]

| Line | Palisade tissue thickness/μm | Spongy tissue  thickness/μm | Palisade/Spongy | Upper epidermal cell thickness/μm | Lower epidermal cell thickness/μm | Leaf thickness/μm | Ratio of palisade/Leaf thickness | Ratio of spongy/Leaf thickness |
| --- | --- | --- | --- | --- | --- | --- | --- | --- |
| WT | 39.84±4.53a | 57.28±7.10a | 0.71±0.14ab | 11.86±1.91a | 11.03±2.38a | 129.14±9.77a | 0.31±0.05a | 0.45±0.06b |
| OE2 | 36.85±3.58b | 50.80±5.03b | 0.73±0.09ab | 12.54±2.23a | 9.51±1.56b | 115.97±9.41b | 0.32±0.04a | 0.44±0.06b |
| *br* | 25.89±2.78c | 39.53±5.70c | 0.67±0.11b | 11.03±1.16b | 8.58±1.29c | 83.62±9.13c | 0.31±0.04a | 0.48±0.09a |
