# Supplementary material for: Characterization and T-DNA insertion sites identification of a multiple-branches mutant br in Betula platyphylla × Betula pendula
Source: BMC Plant Biol. 2019 Nov 12;19:491. doi: 10.1186/s12870-019-2098-y (PMC6852751; doi:10.1186/s12870-019-2098-y)
Supplement: Supplementary file 5 — Additional file 5: DOC 1. The analysis of flanking sequence in br. a: The sequence of T-DNA (pGWB2); b: The sequence of I1L; c: The sequence of I1R; d: The sequence of I2. The sequences in red are mapped to pGWB2, and the sequences in green are mapped to the birch genome in b, c and d. The sequence without a shadow indicates misalignment caused by T-DNA insertion in c. [file 12870_2019_2098_MOESM5_ESM.doc]

a: > pGWB2

TGAGCGTCGCAAAGGCGCTCGGTCTTGCCTTGCTCGTCGGTGATGTACTTCACCAGCTCCGCGAAGTCGCTCTTCTTGATGGAGCGCATGGGGACGTGCTTGGCAATCACGCGCACCCCCCGGCCGTTTTAGCGGCTAAAAAAGTCATGGCTCTGCCCTCGGGCGGACCACGCCCATCATGACCTTGCCAAGCTCGTCCTGCTTCTCTTCGATCTTCGCCAGCAGGGCGAGGATCGTGGCATCACCGAACCGCGCCGTGCGCGGGTCGTCGGTGAGCCAGAGTTTCAGCAGGCCGCCCAGGCGGCCCAGGTCGCCATTGATGCGGGCCAGCTCGCGGACGTGCTCATAGTCCACGACGCCCGTGATTTTGTAGCCCTGGCCGACGGCCAGCAGGTAGGCCGACAGGCTCATGCCGGCCGCCGCCGCCTTTTCCTCAATCGCTCTTCGTTCGTCTGGAAGGCAGTACACCTTGATAGGTGGGCTGCCCTTCCTGGTTGGCTTGGTTTCATCAGCCATCCGCTTGCCCTCATCTGTTACGCCGGCGGTAGCCGGCCAGCCTCGCAGAGCAGGATTCCCGTTGAGCACCGCCAGGTGCGAATAAGGGACAGTGAAGAAGGAACACCCGCTCGCGGGTGGGCCTACTTCACCTATCCTGCCCGGCTGACGCCGTTGGATACACCAAGGAAAGTCTACACGAACCCTTTGGCAAAATCCTGTATATCGTGCGAAAAAGGATGGATATACCGAAAAAATCGCTATAATGACCCCGAAGCAGGGTTATGCAGCGGAAAAGCGCCACGCTTCCCGAAGGGAGAAAGGCGGACAGGTATCCGGTAAGCGGCAGGGTCGGAACAGGAGAGCGCACGAGGGAGCTTCCAGGGGGAAACGCCTGGTATCTTTATAGTCCTGTCGGGTTTCGCCACCTCTGACTTGAGCGTCGATTTTTGTGATGCTCGTCAGGGGGGCGGAGCCTATGGAAAAACGCCAGCAACGCGGCCTTTTTACGGTTCCTGGCCTTTTGCTGGCCTTTTGCTCACATGTTCTTTCCTGCGTTATCCCCTGATTCTGTGGATAACCGTATTACCGCCTTTGAGTGAGCTGATACCGCTCGCCGCAGCCGAACGACCGAGCGCAGCGAGTCAGTGAGCGAGGAAGCGGAAGAGCGCCAGAAGGCCGCCAGAGAGGCCGAGCGCGGCCGTGAGGCTTGGACGCTAGGGCAGGGCATGAAAAAGCCCGTAGCGGGCTGCTACGGGCGTCTGACGCGGTGGAAAGGGGGAGGGGATGTTGTCTACATGGCTCTGCTGTAGTGAGTGGGTTGCGCTCCGGCAGCGGTCCTGATCAATCGTCACCCTTTCTCGGTCCTTCAACGTTCCTGACAACGAGCCTCCTTTTCGCCAATCCATCGACAATCACCGCGAGTCCCTGCTCGAACGCTGCGTCCGGACCGGCTTCGTCGAAGGCGTCTATCGCGGCCCGCAACAGCGGCGAGAGCGGAGCCTGTTCAACGGTGCCGCCGCGCTCGCCGGCATCGCTGTCGCCGGCCTGCTCCTCAAGCACGGCCCCAACAGTGAAGTAGCTGATTGTCATCAGCGCATTGACGGCGTCCCCGGCCGAAAAACCCGCCTCGCAGAGGAAGCGAAGCTGCGCGTCGGCCGTTTCCATCTGCGGTGCGCCCGGTCGCGTGCCGGCATGGATGCGCGCGCCATCGCGGTAGGCGAGCAGCGCCTGCCTGAAGCTGCGGGCATTCCCGATCAGAAATGAGCGCCAGTCGTCGTCGGCTCTCGGCACCGAATGCGTATGATTCTCCGCCAGCATGGCTTCGGCCAGTGCGTCGAGCAGCGCCCGCTTGTTCCTGAAGTGCCAGTAAAGCGCCGGCTGCTGAACCCCCAACCGTTCCGCCAGTTTGCGTGTCGTCAGACCGTCTACGCCGACCTCGTTCAACAGGTCCAGGGCGGCACGGATCACTGTATTCGGCTGCAACTTTGTCATGCTTGACACTTTATCACTGATAAACATAATATGTCCACCAACTTATCAGTGATAAAGAATCCGCGCGTTCAATCGGACCAGCGGAGGCTGGTCCGGAGGCCAGACGTGAAACCCAACATACCCCTGATCGTAATTCTGAGCACTGTCGCGCTCGACGCTGTCGGCATCGGCCTGATTATGCCGGTGCTGCCGGGCCTCCTGCGCGATCTGGTTCACTCGAACGACGTCACCGCCCACTATGGCATTCTGCTGGCGCTGTATGCGTTGGTGCAATTTGCCTGCGCACCTGTGCTGGGCGCGCTGTCGGATCGTTTCGGGCGGCGGCCAATCTTGCTCGTCTCGCTGGCCGGCGCCAGATCTGGGGAACCCTGTGGTTGGCATGCACATACAAATGGACGAACGGATAAACCTTTTCACGCCCTTTTAAATATCCGATTATTCTAATAAACGCTCTTTTCTCTTAGGTTTACCCGCCAATATATCCTGTCAAACACTGATAGTTTAAACTGAAGGCGGGAAACGACAATCTGATCATGAGCGGAGAATTAAGGGAGTCACGTTATGACCCCCGCCGATGACGCGGGACAAGCCGTTTTACGTTTGGAACTGACAGAACCGCAACGTTGAAGGAGCCACTCAGCCGCGGGTTTCTGGAGTTTAATGAGCTAAGCACATACGTCAGAAACCATTATTGCGCGTTCAAAAGTCGCCTAAGGTCACTATCAGCTAGCAAATATTTCTTGTCAAAAATGCTCCACTGACGTTCCATAAATTCCCCTCGGTATCCAATTAGAGTCTCATATTCACTCTCAATCCAAATAATCTGCACCGGATCTGGATCGTTTCGCATGATTGAACAAGATGGATTGCACGCAGGTTCTCCGGCCGCTTGGGTGGAGAGGCTATTCGGCTATGACTGGGCACAACAGACAATCGGCTGCTCTGATGCCGCCGTGTTCCGGCTGTCAGCGCAGGGGCGCCCGGTTCTTTTTGTCAAGACCGACCTGTCCGGTGCCCTGAATGAACTGCAGGACGAGGCAGCGCGGCTATCGTGGCTGGCCACGACGGGCGTTCCTTGCGCAGCTGTGCTCGACGTTGTCACTGAAGCGGGAAGGGACTGGCTGCTATTGGGCGAAGTGCCGGGGCAGGATCTCCTGTCATCTCACCTTGCTCCTGCCGAGAAAGTATCCATCATGGCTGATGCAATGCGGCGGCTGCATACGCTTGATCCGGCTACCTGCCCATTCGACCACCAAGCGAAACATCGCATCGAGCGAGCACGTACTCGGATGGAAGCCGGTCTTGTCGATCAGGATGATCTGGACGAAGAGCATCAGGGGCTCGCGCCAGCCGAACTGTTCGCCAGGCTCAAGGCGCGCATGCCCGACGGCGATGATCTCGTCGTGACCCATGGCGATGCCTGCTTGCCGAATATCATGGTGGAAAATGGCCGCTTTTCTGGATTCATCGACTGTGGCCGGCTGGGTGTGGCGGACCGCTATCAGGACATAGCGTTGGCTACCCGTGATATTGCTGAAGAGCTTGGCGGCGAATGGGCTGACCGCTTCCTCGTGCTTTACGGTATCGCCGCTCCCGATTCGCAGCGCATCGCCTTCTATCGCCTTCTTGACGAGTTCTTCTGAGCGGGACTCTGGGGTTCGAAATGACCGACCAAGCGACGCCCAACCTGCCATCACGAGATTTCGATTCCACCGCCGCCTTCTATGAAAGGTTGGGCTTCGGAATCGTTTTCCGGGACGCCGGCTGGATGATCCTCCAGCGCGGGGATCTCATGCTGGAGTTCTTCGCCCACGGGATCTCTGCGGAACAGGCGGTCGAAGGTGCCGATATCATTACGACAGCAACGGCCGACAAGCACAACGCCACGATCCTGAGCGACAATATGATCGGGCCCGGCGTCCACATCAACGGCGTCGGCGGCGACTGCCCAGGCAAGACCGAGATGCACCGCGATATCTTGCTGCGTTCGGATATTTTCGTGGAGTTCCCGCCACAGACCCGGATGATCCCCGATCGTTCAAACATTTGGCAATAAAGTTTCTTAAGATTGAATCCTGTTGCCGGTCTTGCGATGATTATCATATAATTTCTGTTGAATTACGTTAAGCATGTAATAATTAACATGTAATGCATGACGTTATTTATGAGATGGGTTTTTATGATTAGAGTCCCGCAATTATACATTTAATACGCGATAGAAAACAAAATATAGCGCGCAAACTAGGATAAATTATCGCGCGCGGTGTCATCTATGTTACTAGATCGGGCCTCCTGTCAATGCTGGCGGCGGCTCTGGTGGTGGTTCTGGTGGCGGCTCTGAGGGTGGTGGCTCTGAGGGTGGCGGTTCTGAGGGTGGCGGCTCTGAGGGAGGCGGTTCCGGTGGTGGCTCTGGTTCCGGTGATTTTGATTATGAAAAGATGGCAAACGCTAATAAGGGGGCTATGACCGAAAATGCCGATGAAAACGCGCTACAGTCTGACGCTAAAGGCAAACTTGATTCTGTCGCTACTGATTACGGTGCTGCTATCGATGGTTTCATTGGTGACGTTTCCGGCCTTGCTAATGGTAATGGTGCTACTGGTGATTTTGCTGGCTCTAATTCCCAAATGGCTCAAGTCGGTGACGGTGATAATTCACCTTTAATGAATAATTTCCGTCAATATTTACCTTCCCTCCCTCAATCGGTTGAATGTCGCCCTTTTGTCTTTGGCCCAATACGCAAACCGCCTCTCCCCGCGCGTTGGCCGATTCATTAATGCAGCTGGCACGACAGGTTTCCCGACTGGAAAGCGGGCAGTGAGCGCAACGCAATTAATGTGAGTTAGCTCACTCATTAGGCACCCCAGGCTTTACACTTTATGCTTCCGGCTCGTATGTTGTGTGGAATTGTGAGCGGATAACAATTTCACACAGGAAACAGCTATGACCATGATTACGCCAAGCTTGCATGCCTGCAGGTCCCCAGATTAGCCTTTTCAATTTCAGAAAGAATGCTAACCCACAGATGGTTAGAGAGGCTTACGCAGCAGGTCTCATCAAGACGATCTACCCGAGCAATAATCTCCAGGAAATCAAATACCTTCCCAAGAAGGTTAAAGATGCAGTCAAAAGATTCAGGACTAACTGCATCAAGAACACAGAGAAAGATATATTTCTCAAGATCAGAAGTACTATTCCAGTATGGACGATTCAAGGCTTGCTTCACAAACCAAGGCAAGTAATAGAGATTGGAGTCTCTAAAAAGGTAGTTCCCACTGAATCAAAGGCCATGGAGTCAAAGATTCAAATAGAGGACCTAACAGAACTCGCCGTAAAGACTGGCGAACAGTTCATACAGAGTCTCTTACGACTCAATGACAAGAAGAAAATCTTCGTCAACATGGTGGAGCACGACACACTTGTCTACTCCAAAAATATCAAAGATACAGTCTCAGAAGACCAAAGGGCAATTGAGACTTTTCAACAAAGGGTAATATCCGGAAACCTCCTCGGATTCCATTGCCCAGCTATCTGTCACTTTATTGTGAAGATAGTGGAAAAGGAAGGTGGCTCCTACAAATGCCATCATTGCGATAAAGGAAAGGCCATCGTTGAAGATGCCTCTGCCGACAGTGGTCCCAAAGATGGACCCCCACCCACGAGGAGCATCGTGGAAAAAGAAGACGTTCCAACCACGTCTTCAAAGCAAGTGGATTGATGTGATATCTCCACTGACGTAAGGGATGACGCACAATCCCACTATCCTTCGCAAGACCCTTCCTCTATATAAGGAAGTTCATTTCATTTGGAGAGAACACGGGGGACTCTAGAGTTATCAACAAGTTTGTACAAAAAAGCTGAACGAGAAACGTAAAATGATATAAATATCAATATATTAAATTAGATTTTGCATAAAAAACAGACTACATAATACTGTAAAACACAACATATCCAGTCACTATGGCGGCCCCTCGAGGGGCCGCATTAGGCACCCCAGGCTTTACACTTTATGCTTCCGGCTCGTATAATGTGTGGATTTTGAGTTAGGATCCGGCGAGATTTTCAGGAGCTAAGGAAGCTAAAATGGAGAAAAAAATCACTGGATATACCACCGTTGATATATCCCAATGGCATCGTAAAGAACATTTTGAGGCATTTCAGTCAGTTGCTCAATGTACCTATAACCAGACCGTTCAGCTGGATATTACGGCCTTTTTAAAGACCGTAAAGAAAAATAAGCACAAGTTTTATCCGGCCTTTATTCACATTCTTGCCCGCCTGATGAATGCTCATCCGGAATTCCGTATGGCAATGAAAGACGGTGAGCTGGTGATATGGGATAGTGTTCACCCTTGTTACACCGTTTTCCATGAGCAAACTGAAACGTTTTCATCGCTCTGGAGTGAATACCACGACGATTTCCGGCAGTTTCTACACATATATTCGCAAGATGTGGCGTGTTACGGTGAAAACCTGGCCTATTTCCCTAAAGGGTTTATTGAGAATATGTTTTTCGTCTCAGCCAATCCCTGGGTGAGTTTCACCAGTTTTGATTTAAACGTGGCCAATATGGACAACTTCTTCGCCCCCGTTTTCACCATGGGCAAATATTATACGCAAGGCGACAAGGTGCTGATGCCGCTGGCGATTCAGGTTCATCATGCCGTCTGTGATGGCTTCCATGTCGGCAGAATGCTTAATGAATTACAACAGTACTGCGATGAGTGGCAGGGCGGGGCGTAAAGATCTGGATCCGGCTTACTAAAAGCCAGATAACAGTATGCGTATTTGCGCGCTGATTTTTGCGGTATAAGAATATATACTGATATGTATACCCGAAGTATGTCAAAAAGAGGTGTGCTATGAAGCAGCGTATTACAGTGACAGTTGACAGCGACAGCTATCAGTTGCTCAAGGCATATATGATGTCAATATCTCCGGTCTGGTAAGCACAACCATGCAGAATGAAGCCCGTCGTCTGCGTGCCGAACGCTGGAAAGCGGAAAATCAGGAAGGGATGGCTGAGGTCGCCCGGTTTATTGAAATGAACGGCTCTTTTGCTGACGAGAACAGGGACTGGTGAAATGCAGTTTAAGGTTTACACCTATAAAAGAGAGAGCCGTTATCGTCTGTTTGTGGATGTACAGAGTGATATTATTGACACGCCCGGGCGACGGATGGTGATCCCCCTGGCCAGTGCACGTCTGCTGTCAGATAAAGTCTCCCGTGAACTTTACCCGGTGGTGCATATCGGGGATGAAAGCTGGCGCATGATGACCACCGATATGGCCAGTGTGCCGGTCTCCGTTATCGGGGAAGAAGTGGCTGATCTCAGCCACCGCGAAAATGACATCAAAAACGCCATTAACCTGATGTTCTGGGGAATATAAATGTCAGGCTCCCTTATACACAGCCAGTCTGCAGGTCGACCATAGTGACTGGATATGTTGTGTTTTACAGTATTATGTAGTCTGTTTTTTATGCAAAATCTAATTTAATATATTGATATTTATATCATTTTACGTTTCTCGTTCAGCTTTCTTGTACAAAGTGGTTGATAACAGCGCTTAGAGCTCGAATTTCCCCGATCGTTCAAACATTTGGCAATAAAGTTTCTTAAGATTGAATCCTGTTGCCGGTCTTGCGATGATTATCATATAATTTCTGTTGAATTACGTTAAGCATGTAATAATTAACATGTAATGCATGACGTTATTTATGAGATGGGTTTTTATGATTAGAGTCCCGCAATTATACATTTAATACGCGATAGAAAACAAAATATAGCGCGCAAACTAGGATAAATTATCGCGCGCGGTGTCATCTATGTTACTAGATCGGGAATTAGCTTCATCAACGCAAGACATGCGCACGACCGTCTGACAGGAGAGGAATTTCCGACGAGCACAGAAAGGACTTGCTCTTGGACGTAGGCCTATTTCTCAGGCACATGTATCAAGTGTTCGGACGTGGGTTTTCGATGGTGTATCAGCCGCCGCCAACTGGGAGATGAGGAGGCTTTCTTGGGGGGCAGTCAGCAGTTCATTTCACAAGACAGAGGAACTTGTAAGGAGATGCACTGATTTATCTTGGCGCAAACCAGCAGGACGAATTAGTGGGAATAGCCCGCGAATATCTAAGTTATGCCTGTCGGCATGAGCAGAAACTTCCAATTCGAAACAGTTTGGAGAGGTTGTTTTTGGGCATACCTTTTGTTAGTCAGCCTCTCGATTGCTCATCGTCATTACACAGTACCGAAGTTTGATCGATCTAGTAACATAGATGACACCGCGCGCGATAATTTATCCTAGTTTGCGCGCTATATTTTGTTTTCTATCGCGTATTAAATGTATAATTGCGGGACTCTAATCATAAAAACCCATCTCATAAATAACGTCATGCATTACATGTTAATTATTACATGCTTAACGTAATTCAACAGAAATTATATGATAATCATCGCAAGACCGGCAACAGGATTCAATCTTAAGAAACTTTATTGCCAAATGTTTGAACGATCTGCTTCGACGCACTCCTTCTTTACTCCACCATCTCGTCCTTATTGAAAACGTGGGTAGCACCAAAACGAATCAAGTCGCTGGAACTGAAGTTACCAATCACGCTGGATGATTTGCCAGTTGGATTAATCTTGCCTTTCCCCGCATGAATAATATTGATGAATGCATGCGTGAGGGGTATTTCGATTTTGGCAATAGCTGCAATTGCCGCGACATCCTCCAACGAGCATAATTCTTCAGAAAAATAGCGATGTTCCATGTTGTCAGGGCATGCATGATGCACGTTATGAGGTGACGGTGCTAGGCAGTATTCCCTCAAAGTTTCATAGTCAGTATCATATTCATCATTGCATTCCTGCAAGAGAGAATTGAGACGCAATCCACACGCTGCGGCAACCTTCCGGCGTTCGTGGTCTATTTGCTCTTGGACGTTGCAAACGTAAGTGTTGGATCCCGGTCGGCATCTACTCTATTCCTTTGCCCTCGGACGAGTGCTGGGGCGTCGGTTTCCACTATCGGCGAGTACTTCTACACAGCCATCGGTCCAGACGGCCGCGCTTCTGCGGGCGATTTGTGTACGCCCGACAGTCCCGGCTCCGGATCGGACGATTGCGTCGCATCGACCCTGCGCCCAAGCTGCATCATCGAAATTGCCGTCAACCAAGCTCTGATAGAGTTGGTCAAGACCAATGCGGAGCATATACGCCCGGAGCCGCGGCGATCCTGCAAGCTCCGGATGCCTCCGCTCGAAGTAGCGCGTCTGCTGCTCCATACAAGCCAACCACGGCCTCCAGAAGAAGATGTTGGCGACCTCGTATTGGGAATCCCCGAACATCGCCTCGCTCCAGTCAATGACCGCTGTTATGCGGCCATTGTCCGTCAGGACATTGTTGGAGCCGAAATCCGCGTGCACGAGGTGCCGGACTTCGGGGCAGTCCTCGGCCCAAAGCATCAGCTCATCGAGAGCCTGCGCGACGGACGCACTGACGGTGTCGTCCATCACAGTTTGCCAGTGATACACATGGGGATCAGCAATCGCGCATATGAAATCACGCCATGTAGTGTATTGACCGATTCCTTGCGGTCCGAATGGGCCGAACCCGCTCGTCTGGCTAAGATCGGCCGCAGCGATCGCATCCATGGCCTCCGCGACCGGCTGCAGAACAGCGGGCAGTTCGGTTTCAGGCAGGTCTTGCAACGTGACACCCTGTGCACGGCGGGAGATGCAATAGGTCAGGCTCTCGCTGAATTCCCCAATGTCAAGCACTTCCGGAATCGGGAGCGCGGCCGATGCAAAGTGCCGATAAACATAACGATCTTTGTAGAAACCATCGGCGCAGCTATTTACCCGCAGGACATATCCACGCCCTCCTACATCGAAGCTGAAAGCACGAGATTCTTCGCCCTCCGAGAGCTGCATCAGGTCGGAGACGCTGTCGAACTTTTCGATCAGAAACTTCTCGACAGACGTCGCGGTGAGTTCAGGCTTTTTCATATCGGGGTCGTCCTCTCCAAATGAAATGAACTTCCTTATATAGAGGAAGGGTCTTGCGAAGGATAGTGGGATTGTGCGTCATCCCTTACGTCAGTGGAGATATCACATCAATCCACTTGCTTTGAAGACGTGGTTGGAACGTCTTCTTTTTCCACGATGCTCCTCGTGGGTGGGGGTCCATCTTTGGGACCACTGTCGGCAGAGGCATCTTGAACGATAGCCTTTCCTTTATCGCAATGATGGCATTTGTAGGTGCCACCTTCCTTTTCTACTGTCCTTTTGATGAAGTGACAGATAGCTGGGCAATGGAATCCGAGGAGGTTTCCCGATATTACCCTTTGTTGAAAAGTCTCAATAGCCCTTTGGTCTTCTGAGACTGTATCTTTGATATTCTTGGAGTAGACGAGAGTGTCGTGCTCCACCATGTTGACGGATCTCTAGGACGCGTCCTAGAAGCTAATTCACTGGCCGTCGTTTTACAACGTCGTGACTGGGAAAACCCTGGCGTTACCCAACTTAATCGCCTTGCAGCACATCCCCCTTTCGCCAGCTGGCGTAATAGCGAAGAGGCCCGCACCGATCGCCCTTCCCAACAGTTGCGCAGCCTGAATGGCGCCCGCTCCTTTCGCTTTCTTCCCTTCCTTTCTCGCCACGTTCGCCGGCTTTCCCCGTCAAGCTCTAAATCGGGGGCTCCCTTTAGGGTTCCGATTTAGTGCTTTACGGCACCTCGACCCCAAAAAACTTGATTTGGGTGATGGTTCACGTAGTGGGCCATCGCCCTGATAGACGGTTTTTCGCCCTTTGACGTTGGAGTCCACGTTCTTTAATAGTGGACTCTTGTTCCAAACTGGAACAACACTCAACCCTATCTCGGGCTATTCTTTTGATTTATAAGGGATTTTGCCGATTTCGGAACCACCATCAAACAGGATTTTCGCCTGCTGGGGCAAACCAGCGTGGACCGCTTGCTGCAACTCTCTCAGGGCCAGGCGGTGAAGGGCAATCAGCTGTTGCCCGTCTCACTGGTGAAAAGAAAAACCACCCCAGTACATTAAAAACGTCCGCAATGTGTTATTAAGTTGTCTAAGCGTCAATTTGTTTACACCACAATATATCCTGCCACCAGCCAGCCAACAGCTCCCCGACCGGCAGCTCGGCACAAAATCACCACTCGATACAGGCAGCCCATCAGTCCGGGACGGCGTCAGCGGGAGAGCCGTTGTAAGGCGGCAGACTTTGCTCATGTTACCGATGCTATTCGGAAGAACGGCAACTAAGCTGCCGGGTTTGAAACACGGATGATCTCGCGGAGGGTAGCATGTTGATTGTAACGATGACAGAGCGTTGCTGCCTGTGATCAAATATCATCTCCCTCGCAGAGATCCGAATTATCAGCCTTCTTATTCATTTCTCGCTTAACCGTGACAGGCTGTCGATCTTGAGAACTATGCCGACATAATAGGAAATCGCTGGATAAAGCCGCTGAGGAAGCTGAGTGGCGCTATTTCTTTAGAAGTGAACGTTGACGATATCAACTCCCCTATCCATTGCTCACCGAATGGTACAGGTCGGGGACCCGAAGTTCCGACTGTCGGCCTGATGCATCCCCGGCTGATCGACCCCAGATCTGGGGCTGAGAAAGCCCAGTAAGGAAACAACTGTAGGTTCGAGTCGCGAGATCCCCCGGAACCAAAGGAAGTAGGTTAAACCCGCTCCGATCAGGCCGAGCCACGCCAGGCCGAGAACATTGGTTCCTGTAGGCATCGGGATTGGCGGATCAAACACTAAAGCTACTGGAACGAGCAGAAGTCCTCCGGCCGCCAGTTGCCAGGCGGTAAAGGTGAGCAGAGGCACGGGAGGTTGCCACTTGCGGGTCAGCACGGTTCCGAACGCCATGGAAACCGCCCCCGCCAGGCCCGCTGCGACGCCGACAGGATCTAGCGCTGCGTTTGGTGTCAACACCAACAGCGCCACGCCCGCAGTTCCGCAAATAGCCCCCAGGACCGCCATCAATCGTATCGGGCTACCTAGCAGAGCGGCAGAGATGAACACGACCATCAGCGGCTGCACAGCGCCTACCGTCGCCGCGACCCCGCCCGGCAGGCGGTAGACCGAAATAAACAACAAGCTCCAGAATAGCGAAATATTAAGTGCGCCGAGGATGAAGATGCGCATCCACCAGATTCCCGTTGGAATCTGTCGGACGATCATCACGAGCAATAAACCCGCCGGCAACGCCCGCAGCAGCATACCGGCGACCCCTCGGCCTCGCTGTTCGGGCTCCACGAAAACGCCGGACAGATGCGCCTTGTGAGCGTCCTTGGGGCCGTCCTCCTGTTTGAAGACCGACAGCCCAATGATCTCGCCGTCGATGTAGGCGCCGAATGCCACGGCATCTCGCAACCGTTCAGCGAACGCCTCCATGGGCTTTTTCTCCTCGTGCTCGTAAACGGACCCGAACATCTCTGGAGCTTTCTTCAGGGCCGACAATCGGATCTCGCGGAAATCCTGCACGTCGGCCGCTCCAAGCCGTCGAATCTGAGCCTTAATCACAATTGTCAATTTTAATCCTCTGTTTATCGGCAGTTCGTAGAGCGCGCCGTGCGTCCCGAGCGATACTGAGCGAAGCAAGTGCGTCGAGCAGTGCCCGCTTGTTCCTGAAATGCCAGTAAAGCGCTGGCTGCTGAACCCCCAGCCGGAACTGACCCCACAAGGCCCTAGCGTTTGCAATGCACCAGGTCATCATTGACCCAGGCGTGTTCCACCAGGCCGCTGCCTCGCAACTCTTCGCAGGCTTCGCCGACCTGCTCGCGCCACTTCTTCACGCGGGTGGAATCCGATCCGCACATGAGGCGGAAGGTTTCCAGCTTGAGCGGGTACGGCTCCCGGTGCGAGCTGAAATAGTCGAACATCCGTCGGGCCGTCGGCGACAGCTTGCGGTACTTCTCCCATATGAATTTCGTGTAGTGGTCGCCAGCAAACAGCACGACGATTTCCTCGTCGATCAGGACCTGGCAACGGGACGTTTTCTTGCCACGGTCCAGGACGCGGAAGCGGTGCAGCAGCGACACCGATTCCAGGTGCCCAACGCGGTCGGACGTGAAGCCCATCGCCGTCGCCTGTAGGCGCGACAGGCATTCCTCGGCCTTCGTGTAATACCGGCCATTGATCGACCAGCCCAGGTCCTGGCAAAGCTCGTAGAACGTGAAGGTGATCGGCTCGCCGATAGGGGTGCGCTTCGCGTACTCCAACACCTGCTGCCACACCAGTTCGTCATCGTCGGCCCGCAGCTCGACGCCGGTGTAGGTGATCTTCACGTCCTTGTTGACGTGGAAAATGACCTTGTTTTGCAGCGCCTCGCGCGGGATTTTCTTGTTGCGCGTGGTGAACAGGGCAGAGCGGGCCGTGTCGTTTGGCATCGCTCGCATCGTGTCCGGCCACGGCGCAATATCGAACAAGGAAAGCTGCATTTCCTTGATCTGCTGCTTCGTGTGTTTCAGCAACGCGGCCTGCTTGGCCTCGCTGACCTGTTTTGCCAGGTCCTCGCCGGCGGTTTTTCGCTTCTTGGTCGTCATAGTTCCTCGCGTGTCGATGGTCATCGACTTCGCCAAACCTGCCGCCTCCTGTTCGAGACGACGCGAACGCTCCACGGCGGCCGATGGCGCGGGCAGGGCAGGGGGAGCCAGTTGCACGCTGTCGCGCTCGATCTTGGCCGTAGCTTGCTGGACCATCGAGCCGACGGACTGGAAGGTTTCGCGGGGCGCACGCATGACGGTGCGGCTTGCGATGGTTTCGGCATCCTCGGCGGAAAACCCCGCGTCGATCAGTTCTTGCCTGTATGCCTTCCGGTCAAACGTCCGATTCATTCACCCTCCTTGCGGGATTGCCCCGACTCACGCCGGGGCAATGTGCCCTTATTCCTGATTTGACCCGCCTGGTGCCTTGGTGTCCAGATAATCCACCTTATCGGCAATGAAGTCGGTCCCGTAGACCGTCTGGCCGTCCTTCTCGTACTTGGTATTCCGAATCTTGCCCTGCACGAATACCAGCGACCCCTTGCCCAAATACTTGCCGTGGGCCTCGGCCTGAGAGCCAAAACACTTGATGCGGAAGAAGTCGGTGCGCTCCTGCTTGTCGCCGGCATCGTTGCGCCACATCTAGGTACTAAAACAATTCATCCAGTAAAATATAATATTTTATTTTCTCCCAATCAGGCTTGATCCCCAGTAAGTCAAAAAATAGCTCGACATACTGTTCTTCCCCGATATCCTCCCTGATCGACCGGACGCAGAAGGCAATGTCATACCACTTGTCCGCCCTGCCGCTTCTCCCAAGATCAATAAAGCCACTTACTTTGCCATCTTTCACAAAGATGTTGCTGTCTCCCAGGTCGCCGTGGGAAAAGACAAGTTCCTCTTCGGGCTTTTCCGTCTTTAAAAAATCATACAGCTCGCGCGGATCTTTAAATGGAGTGTCTTCTTCCCAGTTTTCGCAATCCACATCGGCCAGATCGTTATTCAGTAAGTAATCCAATTCGGCTAAGCGGCTGTCTAAGCTATTCGTATAGGGACAATCCGATATGTCGATGGAGTGAAAGAGCCTGATGCACTCCGCATACAGCTCGATAATCTTTTCAGGGCTTTGTTCATCTTCATACTCTTCCGAGCAAAGGACGCCATCGGCCTCACTCATGAGCAGATTGCTCCAGCCATCATGCCGTTCAAAGTGCAGGACCTTTGGAACAGGCAGCTTTCCTTCCAGCCATAGCATCATGTCCTTTTCCCGTTCCACATCATAGGTGGTCCCTTTATACCGGCTGTCCGTCATTTTTAAATATAGGTTTTCATTTTCTCCCACCAGCTTATATACCTTAGCAGGAGACATTCCTTCCGTATCTTTTACGCAGCGGTATTTTTCGATCAGTTTTTTCAATTCCGGTGATATTCTCATTTTAGCCATTTATTATTTCCTTCCTCTTTTCTACAGTATTTAAAGATACCCCAAGAAGCTAATTATAACAAGACGAACTCCAATTCACTGTTCCTTGCATTCTAAAACCTTAAATACCAGAAAACAGCTTTTTCAAAGTTGTTTTCAAAGTTGGCGTATAACATAGTATCGACGGAGCCGATTTTGAAACCACAATTATGGGTGATGCTGCCAACTTACTGATTTAGTGTATGATGGTGTTTTTGAGGTGCTCCAGTGGCTTCTGTGTCTATCAGCTGTCCCTCCTGTTCAGCTACTGACGGGGTGGTGCGTAACGGCAAAAGCACCGCCGGACATCAGCGCTATCTCTGCTCTCACTGCCGTAAAACATGGCAACTGCAGTTCACTTACACCGCTTCTCAACCCGGTACGCACCAGAAAATCATTGATATGGCCATGAATGGCGTTGGATGCCGGGCAACAGCCCGCATTATGGGCGTTGGCCTCAACACGATTTTACGTCACTTAAAAAACTCAGGCCGCAGTCGGTAACCTCGCGCATACAGCCGGGCAGTGACGTCATCGTCTGCGCGGAAATGGACGAACAGTGGGGCTATGTCGGGGCTAAATCGCGCCAGCGCTGGCTGTTTTACGCGTATGACAGTCTCCGGAAGACGGTTGTTGCGCACGTATTCGGTGAACGCACTATGGCGACGCTGGGGCGTCTTATGAGCCTGCTGTCACCCTTTGACGTGGTGATATGGATGACGGATGGCTGGCCGCTGTATGAATCCCGCCTGAAGGGAAAGCTGCACGTAATCAGCAAGCGATATACGCAGCGAATTGAGCGGCATAACCTGAATCTGAGGCAGCACCTGGCACGGCTGGGACGGAAGTCGCTGTCGTTCTCAAAATCGGTGGAGCTGCATGACAAAGTCATCGGGCATTATCTGAACATAAAACACTATCAATAAGTTGGAGTCATTACCCAATTATGATAGAATTTACAAGCTATAAGGTTATTGTCCTGGGTTTCAAGCATTAGTCCATGCAAGTTTTTATGCTTTGCCCATTCTATAGATATATTGATAAGCGCGCTGCCTATGCCTTGCCCCCTGAAATCCTTACATACGGCGATATCTTCTATATAAAAGATATATTATCTTATCAGTATTGTCAATATATTCAAGGCAATCTGCCTCCTCATCCTCTTCATCCTCTTCGTCTTGGTAGCTTTTTAAATATGGCGCTTCATAGAGTAATTCTGTAAAGGTCCAATTCTCGTTTTCATACCTCGGTATAATCTTACCTATCACCTCAAATGGTTCGCTGGGTTTATCGCACCCCCGAACACGAGCACGGCACCCGCGACCACTATGCCAAGAATGCCCAAGGTAAAAATTGCCGGCCCCGCCATGAAGTCCGTGAATGCCCCGACGGCCGAAGTGAAGGGCAGGCCGCCACCCAGGCCGCCGCCCTCACTGCCCGGCACCTGGTCGCTGAATGTCGATGCCAGCACCTGCGGCACGTCAATGCTTCCGGGCGTCGCGCTCGGGCTGATCGCCCATCCCGTTACTGCCCCGATCCCGGCAATGGCAAGGACTGCCAGCGCTGCCATTTTTGGGGTGAGGCCGTTCGCGGCCGAGGGGCGCAGCCCCTGGGGGGATGGGAGGCCCGCGTTAGCGGGCCGGGAGGGTTCGAGAAGGGGGGGCACCCCCCTTCGGCGTGCGCGGTCACGCGCACAGGGCGCAGCCCTGGTTAAAAACAAGGTTTATAAATATTGGTTTAAAAGCAGGTTAAAAGACAGGTTAGCGGTGGCCGAAAAACGGGCGGAAACCCTTGCAAATGCTGGATTTTCTGCCTGTGGACAGCCCCTCAAATGTCAATAGGTGCGCCCCTCATCTGTCAGCACTCTGCCCCTCAAGTGTCAAGGATCGCGCCCCTCATCTGTCAGTAGTCGCGCCCCTCAAGTGTCAATACCGCAGGGCACTTATCCCCAGGCTTGTCCACATCATCTGTGGGAAACTCGCGTAAAATCAGGCGTTTTCGCCGATTTGCGAGGCTGGCCAGCTCCACGTCGCCGGCCGAAATCGAGCCTGCCCCTCATCTGTCAACGCCGCGCCGGGTGAGTCGGCCCCTCAAGTGTCAACGTCCGCCCCTCATCTGTCAGTGAGGGCCAAGTTTTCCGCGAGGTATCCACAACGCCGGCGGCCGCGGTGTCTCGCACACGGCTTCGACGGCGTTTCTGGCGCGTTTGCAGGGCCATAGACGGCCGCCAGCCCAGCGGCGAGGGCAACCAGCCCGG

b: >I1L

CCTATTCCGACGATCTCCACCCTCCGATCTTCCTCGCACCATGTGGATCTCGTCCGCACCGCGTTTGTTTTTATCTCGCTCCGTAGTTCACAGTCCGGTTCGTTTCACTCATAACCGTTGAAGAGTGAGCTCAGATGCGAGTTTATGGTTTTGTTTACGGCACCTCGACCCCAAAAAACTTGATTTGGGTGATGGTTCACGTAGTG

c: >I1R

GTCCGCAATGTGTTATTAAGTTGTCTAAGCGTCAATTTGTTTACAACGGTTACGTCACACCAGAACACTCCAAACCACCGTCCGCGATGTTCAATCTGATACCGGAGGATTGGGGAGGCTACGTCACGCCCTGGGTGAACGAGATCGCCGAGTCGTTCAACTGCCTCAAGTCGCTTCACTTCCGGCGAATGATCGTCAG

d: >I2

ATTCAGGGCACCGGACAGGTCGGTCTTGACAAAAAGAACCGGGCGCCCCTGCGCTGACAGCCGGAACACGGCGGCATCAGAGCAGCCGATTGTCTGTTGTGCCCAGTCATAGCCGAATAGCCTCTCCACCCAAGCGGCCGGAGAACCTGCGTGCAATCCATCTTGTTCAATCATGCGAAACGATCCAGATCCGGTGCAGATTATTTGGATTGAGAGTGAATATGAGACTCTAATTGGATACCGAGGGGAATTTATGGAACGTCAGTGGAGCATTTTTGACAAGAAATATTTGCTAGCTGATAGTGACCTTAGGCGACTTTTGAACGCGCAATAATGGTTTCTGACGTATGTGCTTAGCTCATTAAACTCCAGAAACCCGCGGCTGAGTGGCTCCTTCAACGTTGCGGTTCTGTCAGTTCCAAACGTAAAACGGCTTGTCCCGCGTCATCGGCGGGGGTCATAACGTGACTCCCTTAATGGATTTTCATAAAAAGTAACTTTTTTATCATGTGTGTTTCTTTTGTTTAATTTTTCTGTTTTCTTTTGGGGGTGTGGGGATCAAAGAGTTTCAACCAAAGAGAAGGGCACAACCAAGAAGTCTAGAACTGAAGATTAGCTAACAAACTACGAGATTTTTACAAACAGAAATATGTACACAATAAACAAGAACAGAACAGCTATATTTCACTTCTATTGATATAAAACACAAGAAGACACATGATAAAGTTTCTGTTTCATAATGACGAGACCTTG
